# Causal relationship between inflammatory cytokines and autoimmune thyroid disease: a bidirectional two-sample Mendelian randomization analysis

**DOI:** 10.3389/fimmu.2024.1334772

**Published:** 2024-03-20

**Authors:** Zhiwei Yao, Fengli Guo, Yanlu Tan, Yiyuan Zhang, Yichen Geng, Guang Yang, Song Wang

**Affiliations:** ^1^ Department of Thyroid Surgery, The Affiliated Taian City Central Hospital of Qingdao University, Taian, China; ^2^ Tianjin Medical University General Hospital, Tianjin Medical University, Tianjin, China; ^3^ Department of Thyroid and Breast Surgery, Binzhou Medical University Hospital, Binzhou, China; ^4^ Department of Thyroid and Neck Tumor, Tianjin Medical University Cancer Institute and Hospital, Tianjin, China; ^5^ Department of Interventional Oncology, Zibo Central Hospital, Zibo, China; ^6^ Department of Reproductive Endocrinology, Second Hospital of Shandong University, Jinan, China; ^7^ Nursing College of Binzhou Medical University, Yantai, China

**Keywords:** inflammatory cytokines, autoimmune thyroid disease, causality, GWAS, Mendelian randomization

## Abstract

**Background:**

Autoimmune thyroid disease (AITD) ranks among the most prevalent thyroid diseases, with inflammatory cytokines playing a decisive role in its pathophysiological process. However, the causal relationship between the inflammatory cytokines and AITD remains elusive.

**Methods:**

A two-sample Mendelian randomization (MR) analysis was performed to elucidate the causal connection between AITD and 41 inflammatory cytokines. Genetic variations associated with inflammatory cytokines were sourced from the FinnGen biobank, whereas a comprehensive meta-analysis of genome-wide association studies (GWASs) yielded data on Graves’ disease (GD) and Hashimoto thyroiditis. Regarding the MR analysis, the inverse variance-weighted, MR-Egger, and weighted median methods were utilized. Additionally, sensitivity analysis was conducted using MR-Egger regression, MR-pleiotropy residual sum, and outliers.

**Results:**

Seven causal associations were identified between inflammatory cytokines and AITD. High levels of tumor necrosis factor–β and low levels of stem cell growth factor–β were indicative of a higher risk of GD. In contrast, high levels of interleukin-12p70 (IL-12p70), IL-13, and interferon-γ and low levels of monocyte chemotactic protein–1 (MCP-1) and TNF-α suggested a higher risk of HD. Moreover, 14 causal associations were detected between AITD and inflammatory cytokines. GD increases the levels of macrophage inflammatory protein–1β, MCP-1, monokine induced by interferon-γ (MIG), interferon γ–induced protein 10 (IP-10), stromal cell–derived factor–1α, platelet-derived growth factor BB, β–nerve growth factor, IL-2ra, IL-4, and IL-17 in blood, whereas HD increases the levels of MIG, IL-2ra, IP-10, and IL-16 levels.

**Conclusion:**

Our bidirectional MR analysis revealed a causal relationship between inflammatory cytokines and AITD. These findings offer valuable insights into the pathophysiological mechanisms underlying AITD.

## 1 Introduction

Autoimmune thyroid disease (AITD) encompasses a group of organ-specific autoimmune diseases characterized by the production of antithyroid antibodies and infiltration of lymphocytes in the thyroid gland (1). Epidemiological investigations established that its prevalence is approximately 5% in the general population and has steadily increased in recent years (2, 3). Clinically, Graves’ Disease (GD) and Hashimoto thyroiditis (HT) are the most common forms of AITD, hallmarked by hyperthyroidism and hypothyroidism, respectively (4). Among them, the former represents a specific autoimmune disease featured by increased thyroid hormone secretion driven by an immune system imbalance that promotes the production of thyroid-stimulating antibodies (5). Additionally, as an AITD, the pathogenesis of HT is similar to that of GD. Specifically, the pathogenesis of AITD involves genetic defects and genetic susceptibility, psychological factors, infections, and stress reactions. Meanwhile, environmental pollution and a high-iodine diet can contribute to or exacerbate autoimmune reactions leading to HT (6). At present, there is an urgent need to investigate the pathogenesis of AITD to develop insights into clinical interventions.

Inflammatory cytokines are a group of proteins or peptides generated by immune cells and exhibit a diverse range of biological activities. Consequently, they play a pivotal role in autoimmune responses (7, 8). Previous studies have documented a close correlation between inflammatory cytokines and the development and progression of AITD (9, 10). On the one hand, cytokine imbalance directly or indirectly induces thyroid epithelial cell growth, abnormal differentiation, and immune dysfunction and can also change the state of immunoreactive cells and participate in the occurrence and development of AITD (11). On the other hand, lymphocytes infiltrating the thyroid in patients with AITD can also contribute to cytokine production. For example, in the helper T cell Th1/Th2 subgroups, Th1 cells, predominantly expressing interleukin-2 (IL-2) and interferon-γ (IFN-γ), stimulate delayed hypersensitivity and mediate cellular immune responses. In contrast, Th2 cells, characterized by expression of IL-4, IL-5, IL-10, and IL-13, modulate antibody production and humoral immune responses (12). At the same time, a growing body of evidence has highlighted the pivotal role of Th17 cells in the development of AITD. Of note, Th17 cells predominantly synthesize IL-17A, IL-17F, IL-21, and IL-22, along with their associated cellular and secretory components (13). Although an increasing number of studies have documented an association between inflammatory cytokines and AITD, their causal relationship remains to be investigated.

Given that genetic predisposition plays a key role in the development of AITD, it should be explained from the perspective of genetics (14). Mendelian randomization (MR) is a superior analytical approach for drawing etiological conclusions by employing genetic variation as an instrumental variable (IV) for exposure. This approach has a lower susceptibility to confounding factors owing to the random allocation of germline genetic variation during meiosis, allowing it to accurately represent exposure without being influenced by reverse causation (15). Importantly, MR studies can be performed to explore numerous dependable genetic variants owing to the public release of extensive gene-wide association data. Hence, this investigation aimed to examine the causal relationship between inflammatory cytokines and AITD.

## 2 Materials and methods

### 2.1 Study population

**Figure 1:**
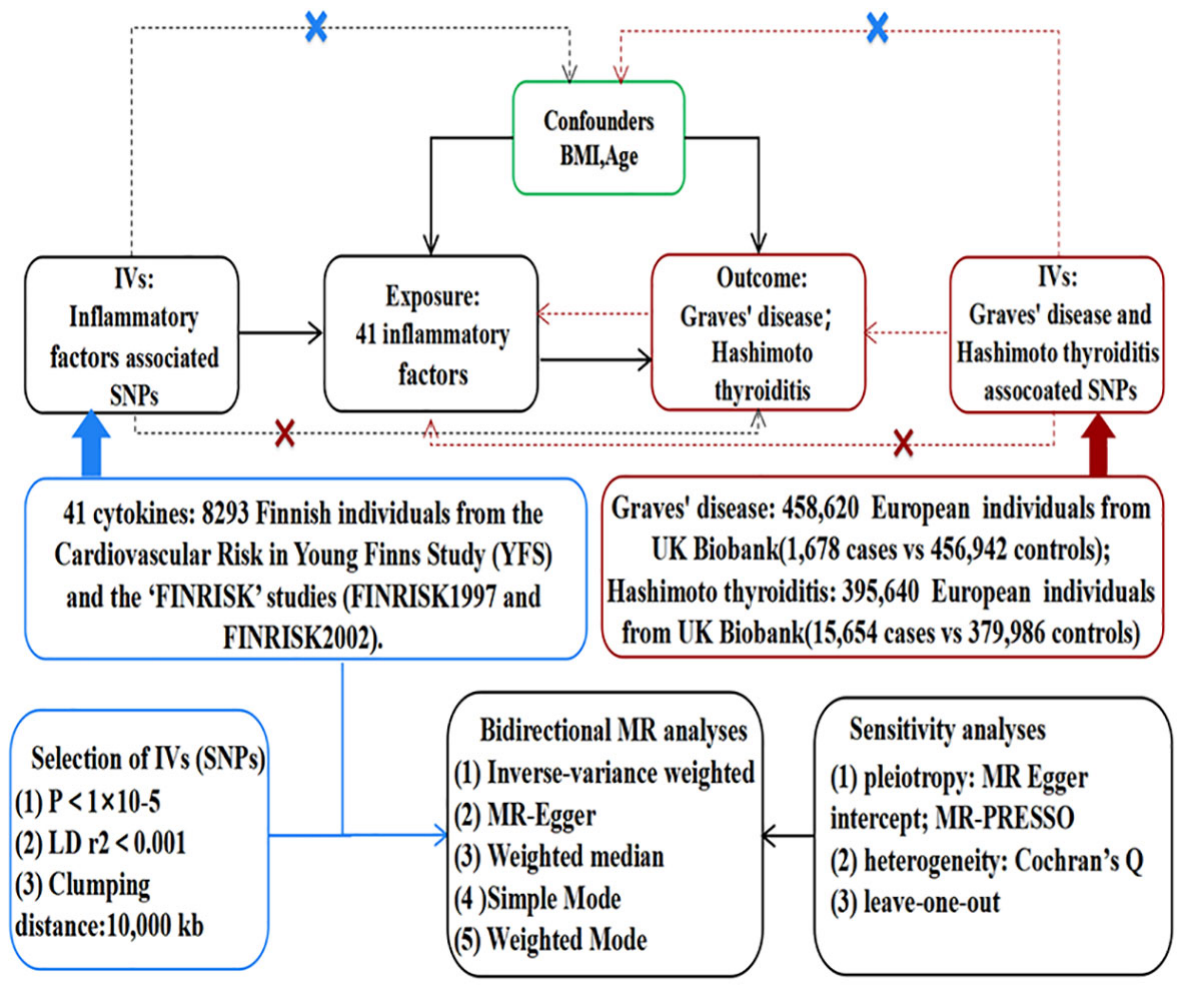
Assumptions and study design of the MR study of the associations between 41 inflammatory factors and autoimmune thyroid disease. BMI, body mass index; IVs, instrumental variables; SNPs, single-nucleotide polymorphisms; MR-PRESSO, Mendelian randomization pleiotropy residual sum and outlier.

As depicted in **Figure 1**, MR relies on three fundamental assumptions: (1) IVs exhibit a robust association with the exposure factor; (2) IVs are not correlated with any confounding factors; and (3) IVs solely impact the results through the exposure and not via any other mechanisms (16). In the current study, pooled data from the published genome-wide association studies (GWASs) of 41 inflammatory cytokines and AITD were utilized. Firstly, genetic variants associated with each inflammatory factor were selected to identify the causal relationship of each inflammatory cytokine with GD and HT. Secondly, AITD-associated genetic variants were employed to infer causality between GD and HT and inflammatory cytokines, respectively.

As detailed in **Table 1**, the genetic associations of 41 inflammatory cytokines were analyzed using data from 8,293 Finnish participants in the Cardiovascular Risk in Finnish Youth Study and the FINRISK Study (17). The first step involved genetic adjustment for 10 principal genetic components, comprising age, gender, and body mass index, as well as for population stratification and cryptic kin using genomic control.

**Table 1:** Details of the studies and datasets used in the study.

Variable	Abbreviation	Ancestry	Population	Consortium
Graves’ disease	GD	European	458,620	IEU GWAS
Hashimoto thyroiditis	HT	European	395,640	IEU GWAS
Macrophage inflammatory protein–1α (CCL3)	MIP-1α	European	3,522	FINRISK 2002 and Young Finns Study
Macrophage inflammatory protein–1β (CCL4)	MIP-1β	European	8,243	FINRISK 1997, FINRISK 2002, and Young Finns Study
Eotaxin (CCL11)	Eotaxin	European	8,153	FINRISK 1997, FINRISK 2002, and Young Finns Study
Monocyte chemotactic protein–1 (CCL2)	MCP-1	European	8,293	FINRISK 1997, FINRISK 2002, and Young Finns Study
Monocyte specific chemokine 3 (CCL7)	MCP-3	European	843	FINRISK 2002 and Young Finns Study
Monokine induced by interferon-γ (CXCL9)	MIG	European	3,685	FINRISK 2002 and Young Finns Study
Interferon γ–induced protein 10 (CXCL10)	IP-10	European	3,685	FINRISK 2002 and Young Finns Study
Cutaneous T-cell attracting (CCL27)	CTACK	European	3,631	FINRISK 2002 and Young Finns Study
Regulated on activation, normal T cell expressed and secreted (CCL5)	RANTES	European	3,421	FINRISK 2002 and Young Finns Study
Growth-regulated oncogene–α	GROα	European	3,505	FINRISK 2002 and Young Finns Study
Stromal cell–derived factor–1α (CXCL12)	SDF-1α	European	5,998	FINRISK 1997, FINRISK 2002, and Young Finns Study
Stem cell growth factor–β	SCGF-β	European	3,682	FINRISK 2002 and Young Finns Study
Platelet-derived growth factor BB	PDGFbb	European	8,293	FINRISK 1997, FINRISK 2002, and Young Finns Study
Stem cell factor	SCF	European	8,290	FINRISK 1997, FINRISK 2002, and Young Finns Study
Granulocyte colony-stimulating factor	GCSF	European	7,904	FINRISK 1997, FINRISK 2002, and Young Finns Study
Vascular endothelial growth factor	VEGF	European	7,118	FINRISK 1997, FINRISK 2002, and Young Finns Study
Hepatocyte growth factor	HGF	European	8,292	FINRISK 1997, FINRISK 2002, and Young Finns Study
Macrophage colony-stimulating factor	MCSF	European	839	FINRISK 2002 and Young Finns Study
β–Nerve growth factor	βNGF	European	3,531	FINRISK 2002 and Young Finns Study
Basic fibroblast growth factor	bFGF	European	7,565	FINRISK 1997, FINRISK 2002, and Young Finns Study
Interleukin-1 receptor antagonist	IL-1ra	European	3,638	FINRISK 2002 and Young Finns Study
Interleukin-1β	IL-1β	European	3,309	FINRISK 2002 and Young Finns Study
Interleukin-2 receptor, α subunit	IL-2ra	European	3,677	FINRISK 2002 and Young Finns Study
Interleukin-2	IL-2	European	3,475	FINRISK 2002 and Young Finns Study
Interleukin-4	IL-4	European	8,214	FINRISK 1997, FINRISK 2002, and Young Finns Study
Interleukin-5	IL-5	European	3,364	FINRISK 2002 and Young Finns Study
Interleukin-6	IL-6	European	8,189	FINRISK 1997, FINRISK 2002, and Young Finns Study
Interleukin-7	IL-7	European	3,409	FINRISK 2002 and Young Finns Study
Interleukin-8 (CXCL8)	IL-8	European	3,526	FINRISK 2002 and Young Finns Study
Interleukin-9	IL-9	European	3,634	FINRISK 2002 and Young Finns Study
Interleukin-10	IL-10	European	7,681	FINRISK 1997, FINRISK 2002, and Young Finns Study
Interleukin-12p70	IL-12p70	European	8,270	FINRISK 1997, FINRISK 2002, and Young Finns Study
Interleukin-13	IL-13	European	3,557	FINRISK 2002 and Young Finns Study
Interleukin-16	IL-16	European	3,483	FINRISK 2002 and Young Finns Study
Interleukin-17	IL-17	European	7,760	FINRISK 1997, FINRISK 2002, and Young Finns Study
Interleukin-18	IL-18	European	3,636	FINRISK 2002, and Young Finns Study
TNF-related apoptosis inducing ligand	TRAIL	European	8,186	FINRISK 1997, FINRISK 2002, and Young Finns Study
Interferon-γ	IFN-γ	European	7,701	FINRISK 1997, FINRISK 2002, and Young Finns Study
Macrophage migration inhibitory factor (glycosylation-inhibiting factor)	MIF	European	3494	FINRISK 2002 and Young Finns Study
Tumor necrosis factor–α	TNF-α	European	3,454	FINRISK 2002 and Young Finns Study
Tumor necrosis factor–β	TNF-β	European	1,559	FINRISK 2002 and Young Finns Study

The GWAS dataset associated with GD and HD was derived from the UK Biobank Project (18). In order to expand the genetic association map beyond European populations, 220 in-depth GWASs were conducted using data from the Biobank in Japan, integrating text mining of past medical histories and electronic medical records. A meta-analysis involving the UK Biobank and FinnGen yielded approximately 5,000 new loci, thereby enhancing the resolution of the human trait genome map. Finally, a statistical decomposition of the overall phenomenon’s summary statistical matrix, coupled with the identification of potential genetic components, assisted in identifying relevant variants and biological mechanisms associated with current disease classifications in the population.

### 2.2 Single-nucleotide polymorphism selection

In the present study, single-nucleotide polymorphisms (SNPs) that are significantly associated with the relative abundance of 41 inflammatory cytokines were selected as IVs. Prior investigations have established that incorporating multiple IVs enhances the interpretation of exposure variation, thereby enhancing the accuracy and reliability of the results. Consequently, the 41 inflammatory cytokines were analyzed on the basis of the outcomes of a significant association analysis with P < 1 × 10^−5^. The linkage disequilibrium criteria were set at r2 < 0.001, whereas the genetic distance was established as 10,000 kb. To ensure the independence of the included SNPs, highly correlated SNPs were excluded. Subsequently, SNPs associated with the relative abundance of the 41 inflammatory cytokines were integrated into the GWAS data of AITD, and corresponding statistical parameters were extracted. The data were harmonized by comparing the statistical parameters at the same sites in the GWAS results of inflammatory cytokines relative abundance and AITD, which facilitated the alignment of the effect values of exposure and outcome with the same effect allele.

### 2.3 Statistical analysis

In the present study, the inverse variance-weighted (IVW), MR-Egger, and weighted median (WME) methods were employed to comprehensively assess causal effects. The IVW approach assumes the validity of all genetic variants. The causal effect value of each individual IV is calculated by IVs using the ratio method. Afterward, each estimate is meticulously summarized for weighted linear regression, yielding the total effect value (19). Regarding regression, the MR-Egger method and the IVW method primarily diverge in their consideration of the intercept term (20). The WME approach utilizes the intermediate impacts of every accessible genetic variation. Estimates were acquired by weighing the inverse variance of each SNP’s association with the outcome (21).

The IVW method is superior to the other two MR methods. Therefore, the IVW method was selected as the preferred method for estimating causal effects. In order to mitigate the risk of false positives, the Benjamini–Hochberg (BH) method was used to control the false discovery rate (FDR). For a clearer explanation of the findings, the impact of the 41 inflammatory cytokines on GD and HT was present as odds ratios (ORs) for each 1 standard deviation genetic predicted change in cytokine levels. In contrast, the impact of GD and HT on the levels of inflammatory cytokines was expressed as β coefficient and 95% confidence intervals (95% CI). Considering that the connection between effect estimates and testing causation might be influenced by weak instrumental biases, the F-statistic was employed to assess the strength of the IVs using the following formula: F = R2 (n − K −1)/k (1 − R2) (22), where R2 represents the variance (per circulating cytokine) explained by the independent variable (IV), and n represents the sample size. R2 was estimated on the basis of the minor allele frequency (MAF) and b-value, calculated using the equation: R2 = 2 × MAF × (1 − MAF) × b2 (23).

Furthermore, to ensure the stability and reliability of the findings, quality control consisting of sensitivity analysis, heterogeneity test, and gene pleiotropy test were carried out at every level. To assess the impact of each SNP on the outcome heterogeneity test, sensitivity analysis was employed using the leave-one-out method, and the combined effect size of the remaining SNPs was determined through sequential deletion of individual SNPs. In the case of significant heterogeneity between IVs (Q_pval < 0.05), the MR effect size was estimated using random-effect IVW. Otherwise, fixed-effect IVW was adopted (24). In order to examine the strength of the findings, additional sensitivity analyses were performed, including MR-Egger regression, MR-pleiotropy residual sum, and outliers (MR-PRESSO) tests. Specifically, WME accurately estimates the causal impact even with less than half of the data originating from the unreliable IV (21). The P-value of the intercept term in MR-Egger regression can serve as an indication of directional pleiotropy (25). Concerning the MR-PRESSO test, the multi-effect effect was corrected by excluding the outlier. In this study, MR analysis and quality control were conducted using R version 4.0.3 and TwoSampleMR version 0.5.6.

## 3 Results

### 3.1 Two-sample MR analysis

The flow chart of 41 inflammatory cytokines and AITD is shown in **Figure 1**. The calculated F-values ranged from 11.156 to 788.955, all meeting the threshold of greater than 10, indicating that weak instrumental bias was unlikely (**Supplementary Tables 1**–**4**). Finally, we identified 21 causal associations between inflammatory cytokines and AITD (**Tables 2**, **3**).

**Table 2:** Effects of the relationship between meaningful inflammatory cytokines and autoimmune thyroid disease in MR analysis.

Exposure	Outcome	SNPs	Methods	OR (95% CI)	*P*-value	*P_FDR_*
TNF-β	GD					
		8	MR-Egger	1.183 (0.982–1.424)	0.127	
		8	Weighted median	1.096 (0.981–1.225)	0.104	
		8	IVW	1.115 (1.024–1.215)	0.013	0.533
SCGF-β	GD					
		24	MR-Egger	0.921 (0.770–1.102)	0.379	
		24	Weighted median	0.989 (0.872–1.121)	0.857	
		24	IVW	0.913 (0.839–0.994)	0.035	0.718
IL-12p70	HD					
		18	MR-Egger	1.117 (0.997–1.251)	0.075	
		18	Weighted median	1.073 (0.990–1.162)	0.086	
		18	IVW	1.088 (1.019–1.162)	0.012	0.342
IL-13	HD					
		18	MR-Egger	1.024 (0.925–1.134)	0.652	
		18	Weighted median	1.059 (0.986–1.136)	0.115	
		18	IVW	1.055 (1.002–1.111)	0.041	0.385
IFN-γ	HD					
		12	MR-Egger	1.111 (0.871–1.416)	0.416	
		12	Weighted median	1.106 (0.935–1.308)	0.239	
		12	IVW	1.150 (1.018–1.299)	0.025	0.342
MCP-1	HD					
		27	MR-Egger	1.087 (0.924–1.278)	0.322	
		27	Weighted median	0.917 (0.823–1.021)	0.113	
		27	IVW	0.930 (0.866–0.999)	0.047	0.385
TNF-α	HD					
		10	MR-Egger	0.763 (0.640–0.911)	0.017	
		10	Weighted median	0.887 (0.785–1.003)	0.056	
		10	IVW	0.892 (0.812–0.981)	0.018	0.342

MR, Mendelian randomization analysis; SNPs, number of single-nucleotide polymorphism; CI, confidence interval; OR, odds ratio; 95% CI, 95% confidence interval; P_FDR_, P-value was calculated by the Benjamini–Hochberg method; GD, Graves’ disease; HT, Hashimoto thyroiditis; IVW, inverse variance-weighted.

**Table 3:** Effects of the relationship between autoimmune thyroid disease and meaningful inflammatory cytokines in reverse MR analysis.

Exposure	Outcome	SNPs	Methods	Beta (95% CI)	*P*-value	*P_FDR_*
GD	MIP-1β					
		22	MR-Egger	0.079 (−0.032–0.191)	0.177	
		22	Weighted median	0.055 (−0.001–0.111)	0.054	
		22	IVW	0.049 (0.010–0.088)	0.014	0.131
GD	MCP-1					
		22	MR-Egger	0.055 (−0.049–0.159)	0.316	
		22	Weighted median	0.063 (0.011–0.114)	0.017	
		22	IVW	0.055 (0.019–0.092)	0.003	0.082
GD	MIG					
		22	MR-Egger	0.250 (0.076–0.424)	0.011	
		22	Weighted median	0.048 (−0.032–0.128)	0.242	
		22	IVW	0.080 (0.014–0.146)	0.018	0.131
GD	IP-10					
		22	MR-Egger	0.180 (−0.011–0.371)	0.080	
		22	Weighted median	0.049 (−0.038–0.136)	0.269	
		22	IVW	0.076 (0.008–0.145)	0.028	0.131
GD	SDF-1α					
		22	MR-Egger	0.021 (−0.089–0.130)	0.716	
		22	Weighted median	0.060 (0.006–0.113)	0.029	
		22	IVW	0.042 (0.004–0.080)	0.030	0.131
GD	PDGFbb					
		22	MR-Egger	−0.005 (−0.109–0.099)	0.927	
		22	Weighted median	0.047 (−0.006–0.099)	0.081	
		22	IVW	0.052 (0.015–0.089)	0.006	0.082
GD	βNGF					
		22	MR-Egger	0.105 (−0.068–0.279)	0.249	
		22	Weighted median	0.140 (0.058–0.222)	0.001	
		22	IVW	0.066 (0.006–0.126)	0.032	0.131
GD	IL-2ra					
		22	MR-Egger	0.134 (−0.021–0.289)	0.106	
		22	Weighted median	0.045 (−0.031–0.122)	0.247	
		22	IVW	0.062 (0.007–0.117)	0.026	0.131
GD	IL-4					
		22	MR-Egger	0.057 (−0.049–0.162)	0.304	
		22	Weighted median	0.068 (0.017–0.119)	0.009	
		22	IVW	0.054 (0.017–0.092)	0.004	0.082
GD	IL-17					
		22	MR-Egger	0.050 (−0.061–0.160)	0.389	
		22	Weighted median	0.056 (0.001–0.110)	0.045	
		22	IVW	0.044 (0.006–0.082)	0.025	0.131
HD	MIG					
		22	MR-Egger	0.393 (0.052–0.735)	0.050	
		22	Weighted median	0.152 (0.010–0.295)	0.037	
		22	IVW	0.234 (0.109–0.359)	2.35E-4	0.010
HD	IL-2ra					
		22	MR-Egger	0.214 (−0.070–0.498)	0.175	
		22	Weighted median	0.100 (−0.033–0.234)	0.141	
		22	IVW	0.136 (0.035–0.236)	0.008	0.085
HD	IP-10					
		22	MR-Egger	0.100 (−0.273–0.472)	0.613	
		22	Weighted median	0.150 (0.010–0.290)	0.036	
		22	IVW	0.183 (0.052–0.315)	0.006	0.085
HD	IL-16					
		22	MR-Egger	0.181 (−0.066–0.428)	0.185	
		22	Weighted median	0.134 (0.009–0.259)	0.036	
		22	IVW	0.124 (0.034–0.213)	0.007	0.085

MR, Mendelian randomization analysis; SNPs, number of single-nucleotide polymorphism; CI, confidence interval; 95% CI, 95% confidence interval; P_FDR_, P-value was calculated by the Benjamini–Hochberg method; GD, Graves’ disease; HT, Hashimoto thyroiditis; IVW, inverse variance-weighted.

### 3.2 Inflammatory cytokines and autoimmune thyroid disease

**Figure 2:**
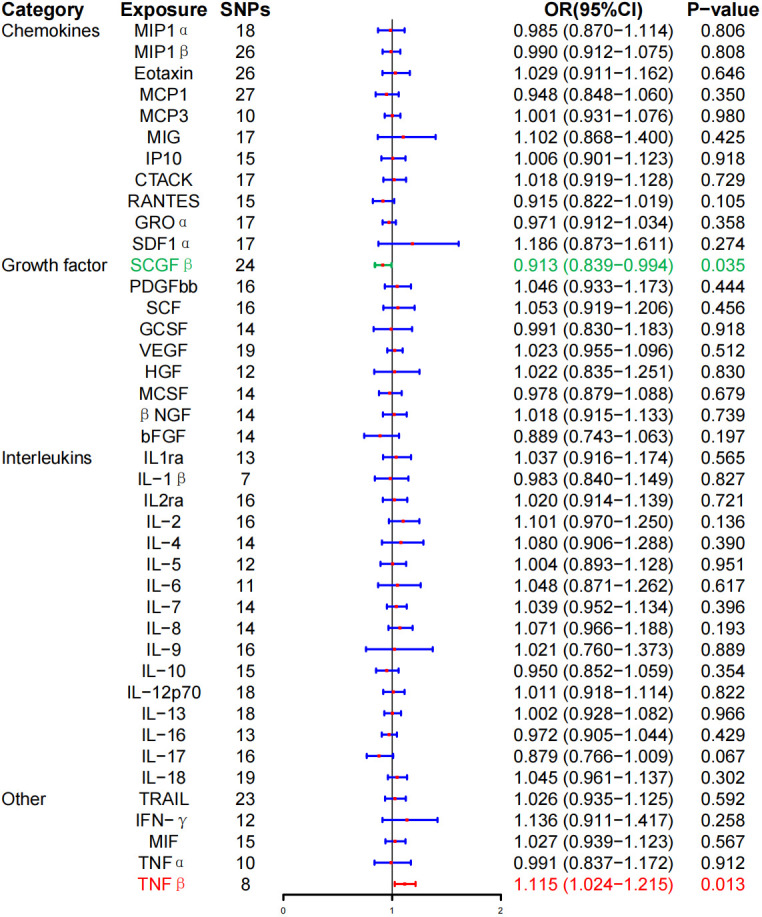
Associations between genetically predicted inflammatory cytokines and Graves’ disease. SNPs, single-nucleotide polymorphisms; OR, odds ratio; 95% CI, 95% confidence interval; red means positive correlation; green means negative correlation.

In the MR analysis, the level of TNF-β was positively correlated with the risk of GD (OR, 1.115; 95% CI, 1.024–1.215; P = 0.013). However, the level of stem cell growth factor–β (SCGF-β) was negatively correlated with the risk of GD (OR, 0.913; 95% CI, 0.839–0.994; P = 0.035). Interestingly, no correlation was noted between the levels of inflammatory cytokines and GD following BH correction (**Figure 2**; **Table 2**).

Furthermore, the WME method did not detect a significant correlation between the level of tumor necrosis factor–β (TNF-β) (OR, 1.096; 95% CI, 0.981–1.225; P = 0.104) and SCG-β (OR, 0.989; 95% CI, 0.872–1.121; P = 0.857) on the risk of GD, but the direction of the effect was consistent with that of IVW (**Table 2**; **Supplementary Figure 1**). As anticipated, the MR-Egger regression intercept showed no evidence of pleiotropy between TNF-β, SCGF-β, and GD (intercept P = 0.511 for TNF-β and intercept P = 0.915 for SCGF-β). No outliers were detected by MR-PRESSO regression. Heterogeneity and sensitivity analysis results corroborated the accuracy of the results (**Table 4**). Lastly, the leave-one-out method further validated the robustness of the data (**Supplementary Figure 5**).

**Table 4:** Heterogeneity and sensitivity analysis of meaningful inflammatory cytokines and risk of autoimmune thyroid disease.

Exposure	Outcome	Methods	Q	*P*	Intercept	*P*	MR-PRESSO
TNF-β	GD						
		IVW	5.795	0.564	−0.015	0.511	0.650
		MR-Egger	5.307	0.505			
SCGF-β	GD						
		IVW	25.219	0.339	−0.002	0.915	0.308
		MR-Egger	25.206	0.287			
IL-12p70	HT						
		IVW	6.863	0.976	−0.024	0.656	0.554
		MR-Egger	6.657	0.966			
IL-13	HT						
		IVW	19.306	0.253	0.023	0.759	0.903
		MR-Egger	19.182	0.206			
IFN-γ	HT						
		IVW	16.508	0.086	0.084	0.533	0.759
		MR-Egger	15.771	0.072			
MCP-1	HT						
		IVW	24.352	0.499	0.067	0.330	0.856
		MR-Egger	23.363	0.498			
TNF-α	HT						
		IVW	8.307	0.504	−0.067	0.444	0.389
		MR-Egger	7.658	0.468			

MR-PRESSO, Mendelian randomization pleiotropy residual sum and outlier; GD, Graves’ disease; HT, Hashimoto thyroiditis; IVW, inverse variance-weighted.

**Figure 3:**
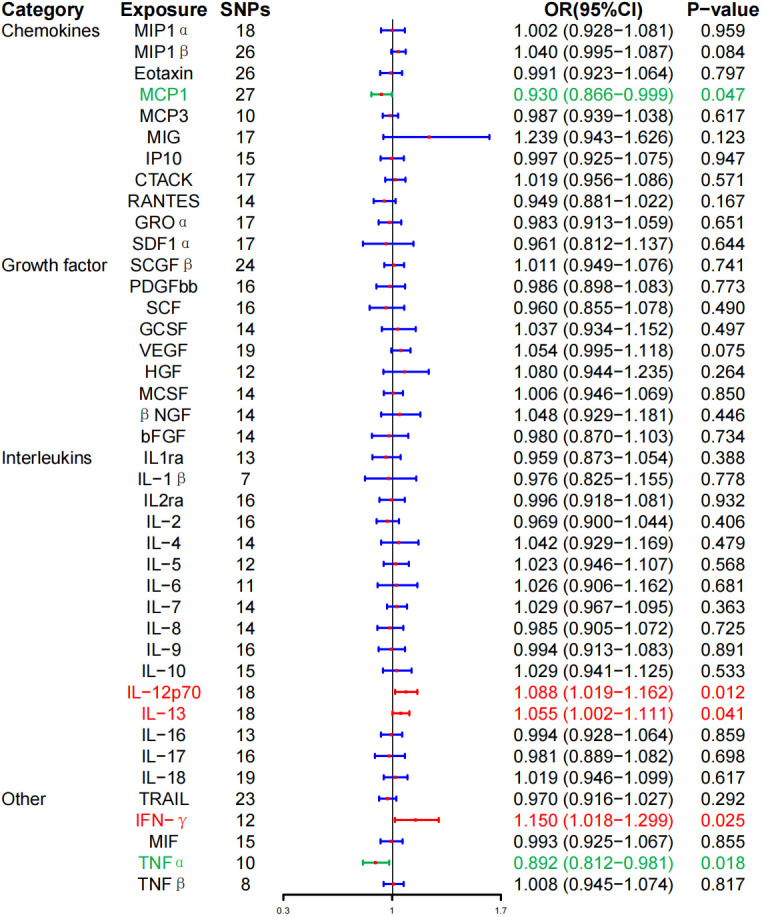
Associations between genetically predicted inflammatory cytokines and Hashimoto thyroiditis. SNPs, single-nucleotide polymorphisms; OR, odds ratio; 95% CI, 95% confidence interval; red means positive correlation; green means negative correlation.

At the same time, high levels of IL-12p70 (OR, 1.088; 95% CI, 1.019–1.162, P = 0.012), IL-13 (OR, 1.055; 95% CI, 1.002–1.111, P = 0.041), and IFN-γ (OR, 1.150; 95% CI, 1.018–1.299; P = 0.025) were positively associated with the risk of HD. Whereas, high levels of monocyte chemotactic protein–1 (MCP-1) (OR, 0.930; 95% CI, 0.866–0.999; P = 0.047) and TNF-α (OR, 0.892; 95% CI, 0.812–0.981; P = 0.018) were negatively associated with the risk of HD, and no correlation between inflammatory cytokines and HD was found after BH correction (**Figure 3**; **Table 2**).

WME method did not detect IL-12p70 (OR, 1.073; 95% CI, 0.990–1.162; P = 0.086), IL-13 (OR, 1.059; 95% CI, 0.9866–1.136, P = 0.115), IFN-γ (OR, 1.106; 95% CI, 0.935–1.308; P = 0.239), MCP-1 (OR, 0.917; 95% CI, 0.823–1.021; P = 0.113), TNF-α (OR, 0.887; 95% CI, 0.7855–1.003; P = 0.056) had a significant effect on the risk of HD, but the direction of effect was consistent with that of IVW (**Table 2**; **Supplementary Figure 2**). The MR-Egger regression intercept showed no evidence of pleiotropy between these inflammatory cytokines and AITD (intercept P = 0.656 for IL-12p70, intercept P = 0.759 for IL-13, intercept P = 0.533 for IFN-γ, intercept P = 0.330 for MCP-1, and intercept P = 0.444 for TNF-α). No outliers were detected by MR-PRESSO regression. Heterogeneity analysis results confirmed the accuracy of the results (**Table 4**). At the same time, leave-one-out method further verified the robustness of the data (**Supplementary Figure 6**).

### 3.3 Autoimmune thyroid diseases and inflammatory cytokines

**Figure 4:**
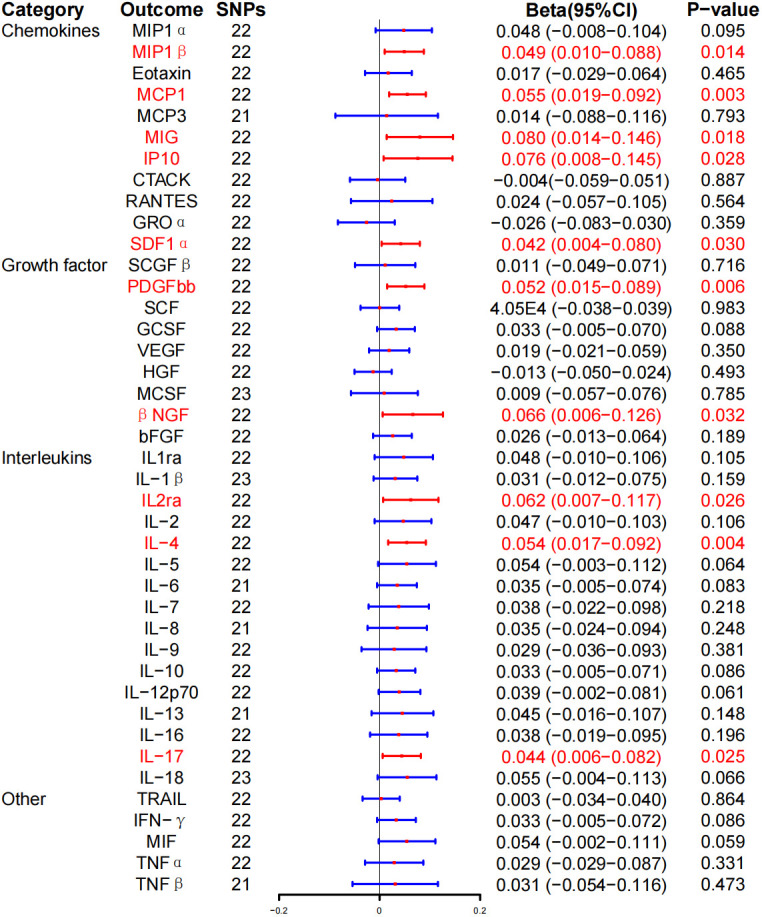
Associations between genetically predicted Graves’ disease and inflammatory cytokines. SNPs, single-nucleotide polymorphisms; 95% CI, 95% confidence interval; red means positive correlation.

In reverse MR analysis, IVW method revealed that GD is associated with higher levels of macrophage inflammatory protein–1β (MIP-1β) [Beat (β), 0.049; 95% CI, 0.010–0.088; P = 0.014], MCP-1 (β, 0.055; 95% CI, 0.019–0.092; P = 0.003), monokine induced by interferon-γ (MIG) (β, 0.080; 95% CI, 0.014–0.146; P = 0.018), interferon γ–induced protein 10 (IP-10) (β, 0.076; 95% CI, 0.008–0.145; P = 0.028), stromal cell–derived factor–1α (SDF-1α) (β, 0.042; 95% CI, 0.004–0.080; P = 0.030), platelet-derived growth factor BB (PDGFbb) (β, 0.052; 95% CI, 0.015–0.089; P = 0.006), β–nerve growth factor (βNGF) (β, 0.066; 95% CI, 0.006–0.126; P = 0.032), IL-2ra (β, 0.062; 95% CI, 0.007–0.117; P = 0.026), IL-4 (β, 0.054; 95% CI, 0.017–0.092; P = 0.004), and IL-17 (β, 0.044; 95% CI, 0.006–0.082; P = 0.025). On the other hand, no correlation was found between GD and inflammatory cytokines after BH correction (**Figure 4**; **Table 3**).

WME exposed that the levels of MIP-1β (β, 0.055; 95% CI, 0.001–0.111; P = 0.054), MIG (β, 0.048; 95% CI, 0.032–0.128; P = 0.242), IP-10 (β, 0.049; 95% CI, 0.038–0.136; P = 0.269), PDGFbb (β, 0.047; 95% CI, 0.006–0.099; P = 0.081), and IL-2ra (β, 0.045; 95% CI, −0.031–0.122; P = 0.247) were significantly associated with the risk of GD. Similarly, there was a positive correlation between the risk and GD and the levels of MCP-1 (β, 0.063; 95% CI, 0.011–0.114; P = 0.017), SDF-1α (β, 0.060; 95% CI, 0.006–0.113; P = 0.029), βNGF (β, 0.140; 95% CI, 0.058–0.222; P = 0.001), IL-4 (β, 0.068; 95% CI, 0.017–0.119, P = 0.009), and IL-17 (β, 0.056; 95% CI = 0.001–0.110, P = 0.045) (**Table 3**; **Supplementary Figure 3**). At the same time, MR-Egger regression analysis displayed no evidence of directional pleiotropy (intercept P = 0.570 for MIP-1β, intercept P = 0.986 for MCP-1, intercept P = 0.054 for MIG, intercept P = 0.270 for IP-10, intercept P = 0.684 for SDF-1α, intercept P = 0.986 for MCP-1, intercept P = 0.054 for MIG, intercept P = 0.270 for IP-10, intercept P = 0.533 for PDGFbb, intercept P = 0.330 for βNGF, intercept P = 0.533 for IL-2ra, intercept P = 0.330 for IL-4, and intercept P = 0.444 for IL-17). No outliers were detected by MR-PRESSO regression. Heterogeneity analysis results confirmed the accuracy of the results (**Table 5**), whereas the leave-one-out method verified the robustness of the data (**Supplementary Figure 7**).

**Table 5:** Heterogeneity and sensitivity analysis of autoimmune thyroid disease with meaningful inflammatory cytokines.

Exposure	Outcome	Methods	Q	*P*	Intercept	*P*	MR-PRESSO
GD	MIP-1β						
		IVW	23.261	0.330	−0.007	0.570	0.340
		MR-Egger	22.880	0.295			
GD	MCP-1						
		IVW	16.927	0.716	1.98E-4	0.986	0.650
		MR-Egger	16.926	0.658			
GD	MIG						
		IVW	30.558	0.081	−0.038	0.054	0.050
		MR-Egger	25.248	0.192			
GD	IP-10						
		IVW	32.345	0.054	−0.023	0.270	0.070
		MR-Egger	30.391	0.064			
GD	SDF-1α						
		IVW	20.976	0.460	0.005	0.684	0.510
		MR-Egger	20.799	0.409			
GD	PDGFbb						
		IVW	14.509	0.847	0.013	0.266	0.800
		MR-Egger	13.198	0.869			
GD	βNGF						
		IVW	24.186	0.284	−0.009	0.642	0.200
		MR-Egger	23.920	0.246			
GD	IL-2ra						
		IVW	19.698	0.540	−0.016	0.345	0.560
		MR-Egger	18.764	0.537			
GD	IL-4						
		IVW	15.716	0.785	−0.001	0.965	0.830
		MR-Egger	15.714	0.734			
GD	IL-17						
		IVW	21.125	0.451	−0.001	0.911	0.460
		MR-Egger	21.112	0.391			
HT	MIG						
		IVW	20.587	0.024	−0.030	0.353	0.089
		MR-Egger	18.603	0.029			
HT	IL-2ra						
		IVW	13.292	0.208	−0.015	0.576	0.160
		MR-Egger	12.814	0.171			
HT	IP-10						
		IVW	22.664	0.012	0.016	0.647	0.510
		MR-Egger	22.113	0.009			
HT	IL-16						
		IVW	9.443	0.491	−0.011	0.640	0.440
		MR-Egger	9.203	0.419			

MR-PRESSO, Mendelian randomization pleiotropy residual sum and outlier; GD, Graves’ disease; HT, Hashimoto thyroiditis; IVW, inverse variance-weighted.

**Figure 5:**
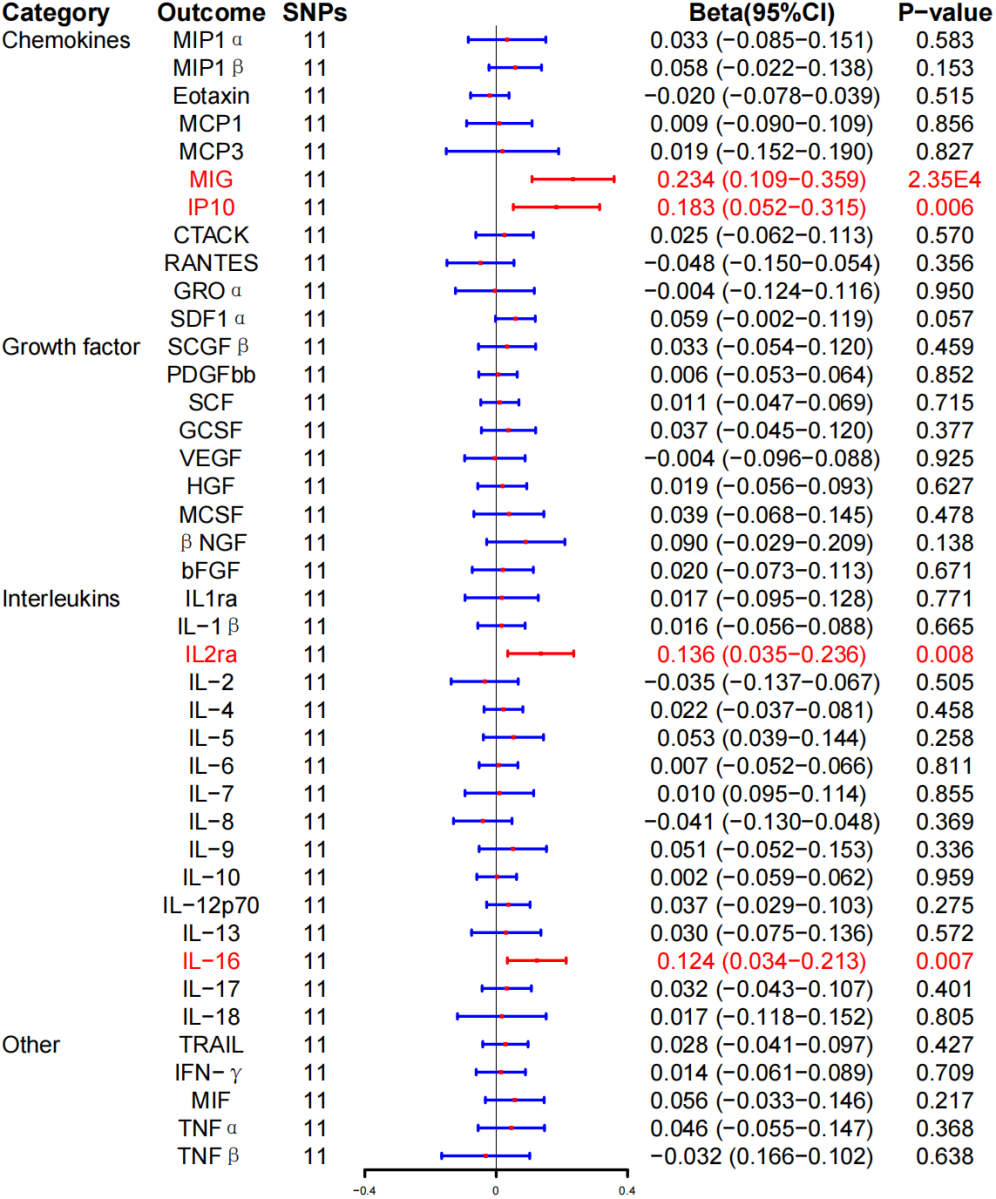
Associations between genetically predicted Hashimoto thyroiditis and inflammatory cytokines. SNPs, single-nucleotide polymorphisms; 95% CI, 95% confidence interval; red means positive correlation.

In addition, we found that HD induced MIG (β, 0.234; 95% CI, 0.109–0.359; P = 2.35E-4), IL-2ra (β, 0.136; 95% CI, 0.035–0.236; P = 0.008), IP-10 (β, 0.183; 95% CI, 0.052–0.315; P = 0.006), and IL-16 (β, 0.124; 95% CI, 0.034–0.213; P = 0.007) increased levels. However, after BH correction, there was a significant positive correlation between HD risk and MIG expression level (P_FDR_ = 0.010) (**Figure 5**; **Table 3**).

WME method showed that the risk of HD was associated with serum MIG (β, 0.152; 95% CI, 0.010–0.295; P = 0.037), IP-10 (β, 0.150; 95% CI, 0.010–0.290; P = 0.036), and IL-16 (β, 0.134; 95% CI, 0.009–0.259, P = 0.036) expression level was positively correlated with HD. No correlation was detected between HD risk and IL-2ra (β, 0.100; 95% CI, −0.033–0.234; P = 0.141) expression levels (**Table 3**; **Supplementary Figure 4**). In addition, the MR-Egger regression intercept showed no evidence of pleiotropy between HD and these circulating cytokines (intercept P = 0.353 for MIG, intercept P = 0.576 for IL-2ra, intercept P = 0.647 for IP-10, and intercept P = 0.640 for IL-16). No outliers were detected by MR-PRESSO regression. Heterogeneity analysis results confirmed the accuracy of the results (**Table 5**). At the same time, the remaining result further verified the robustness of the data (**Supplementary Figure 8**).

## 4 Discussion

This study utilized MR to investigate the causal connection between AITD and inflammatory cytokines. To the best of our knowledge, this is the first study to uncover a causal association between the levels of circulating cytokines and AITD. Specifically, high levels of circulating TNF-β and low levels of SCGF-β were associated with a higher risk of GD. TGF-β belongs to the growth and transformational growth family (26) and plays a key role in the pathogenesis of GD (27, 28). In patients with GD, the TGF-β–stimulated humoral response of Th2 cells to infiltrate the thyroid gland leads to the synthesis of thyroid-stimulating hormone receptor (TSH) autoantibodies (TRAb), thereby promoting the development of GD (29–31). SCGF-β plays a vital role in promoting hematopoiesis and hematopoietic recovery. In tissues and organs beyond the bone marrow, SCGF-β can rapidly activate dormant stem cells and stimulate their growth, simultaneously regulating the internal microenvironment to create favorable growth conditions for stem cells (32, 33). Therefore, we postulate that the decrease in the level of SCGF-β may be associated with the inhibition of thyroid stem cell activity in patients with GD. Concurrently, patients with high circulating levels of IL-12p70, IL-13, and IFN-γ and low levels of MCP-1 and TNF-α are at a higher risk of developing HD. IL-12p70, also referred to as natural killer (NK) cell–stimulating factor, serves as a ligand for IL-12 (34). Its primary immunomodulatory function lies in inducing the differentiation of early helper T cells into Th1 cells, which, in turn, generate pro-inflammatory proteins that trigger macrophage activation, ultimately leading to the initiation of cytotoxic effects (35). Earlier studies have established that IL-12 can exacerbate cytotoxic effects in mice with autoimmune thyroiditis by eliciting a Th1 immune response (36). IL-13, a member of the interleukin family, is largely secreted by activated Th2 cells and plays a critical role in allergic inflammation (37). It promotes M2 polarization. Furthermore, IL-13 actively contributes to the survival, activation, and recruitment of eosinophils (38). Presently, studies investigating the association between IL-13 and AITD are scarce. Therefore, it is essential to undertake comprehensive studies in the future to elucidate this relationship. IFN-γ is the sole member of type II interferons and is produced not only by T cells, macrophages, and NK cells but also by thyroid cells (39). Its immunomodulatory effects include increased phagocytosis of macrophages, NK cell activation, enhancing the expression level of its antigens, and the toxicity of sensitized lymphocytes on target cells (9). Animal studies have documented that IFN-γ–induced apoptosis of thyroid cells is implicated in the pathogenesis of HT (40). Clinical studies further unveiled a significant rise in the number of IFN-γ+ cells in patients with HT (41).

MCP-1 belongs to the CC chemokine family and plays a fundamental role in the inflammatory process (42). Currently, numerous clinical studies have observed an elevated expression level of MCP-1 in both thyroid tissue and serum of patients with AITD (43, 44). Importantly, our study found that low levels of MCP-1 were associated with a higher risk of HD, whereas reverse MR analysis revealed up-regulated MCP-1 expression in patients with GD. TNF-α plays an instrumental role in modulating the inflammatory response, apoptosis, and immune cell activity. Additionally, it triggers the transcription and expression of a diverse array of cytokines, thereby facilitating their synthesis and release (45). Given its central role in the inflammatory response and immune regulation, researchers have developed numerous drugs targeting TNF-α for conditions such as rheumatoid arthritis (46) and Crohn’s disease (47). Notably, our study detected a negative association between TNF-α levels and HD. Further investigations with more extensive databases are warranted to validate this correlation.

Reverse MR analysis showed that GD can increase the blood levels of MIP-1β, MCP-1, MIG, IP-10, SDF-1α, PDGFbb, βNGF, IL-2ra, IL-4, and IL-17, whereas HD can increase the blood levels of MIG, IL-2ra, IP-10, and IL-16. After BH correction, a strong positive correlation was identified between the risk of HD and the expression level of MIG. MIP-1β is a CC chemokine that selectively binds to the CCR5 receptor. It chemotaxes NK cells, monocytes, and various other immune cells and can be generated by mast cells, endothelial cells, macrophages, and CD8+ T cells (48). Kemp et al. (46) observed an increased expression of MIP-1β in the thyroid tissue of patients with AITD, thereby confirming the significant role of MIP-1 in pro-inflammatory processes. MIG belongs to the glutamic acid-leucine-arginine (ELR)-negative CXC chemokine subfamily and can be triggered by IFN-γ (49). CXCL9 plays an instrumental role in modulating the immune system and promoting inflammation. Of note, the recruitment, transport, and maintenance of particular subgroups of activated lymphocytes are essential for initiating and sustaining AITD (50). The secretion of chemokines that bind to CXCR3 by thyroid cells is stimulated by IFN-γ, subsequently attracting Th1 lymphocytes that express CXCR3 and secreting IFN-γ (51). Disrupting MIG could alleviate inflammatory reactions. Therefore, we postulate that targeting MIG may constitute a therapeutic target for the treatment of AITD. IP-10 is a member of the CXC chemokine family (52) and shares receptors such as CXCR3 with MIG. Inflammatory cells, attracted by IP-10, include chemotactic T lymphocytes that infiltrate and proliferate, thereby mediating antibody-specific autoimmune responses and resulting in gland destruction (53). Romagnani et al. (54) documented that the expression of IP-10, MIG, and CXCR3 was downregulated or absent in healthy thyroid tissue, whereas chemokines and their receptors were abundant in the thyroid glands of the majority of patients with GD. IP-10 and MIG were localized in infiltrating lymphocytes, macrophages, and resident epithelial follicular cells, whereas CXCR3 was predominantly localized in infiltrating inflammatory cells. SDF-1α belongs to the α subfamily of chemokines. Acting as a ligand, it binds to the CXCR4 receptor. The SDF-1α/CXCR4 signaling pathway plays an essential role in various physiological processes, including cell transport, angiogenesis, embryogenesis, tumor invasion, and metastasis (55, 56). Consequently, existing studies predominantly focused on investigating the value of SDF-1α as a therapeutic target in thyroid malignancies. However, research addressing its role in AITD is limited (57, 58). PDGFbb participates in regulating cell growth and division. Following injury, platelets secrete PDGFbb, which facilitates the infiltration of neutrophils and macrophages. Simultaneously, PDGFbb promotes the secretion of a new extracellular matrix by fibroblasts and conduces to Insulin-like growth factor (IGF)-1–mediated reepithelialization (59, 60). Previous investigations have evinced that PDGFbb possesses the capability to induce orbital fibroblast proliferation, as well as the production of hyaluronic acid and cytokines in the orbital tissue of patients with Graves’ ophthalmopathy (61). βNGF primarily stimulates the growth, development, differentiation, and maturation of both central and peripheral neurons, concurrently upholding the physiological functioning of the nervous system (62). Simultaneously, it is involved in immunomodulation, directly influencing the innate and adaptive immune responses of B and T cells. Importantly, it induces the release of neuropeptides and neurotransmitters, thereby modulating immune system activation within inflammatory tissues (63). It is worthwhile mentioning that elevated levels of autoantibodies against βNGF were noted in the blood and tissues of individuals with AITD. Moreover, it has the potential to activate mast cells, triggering the release of inflammatory mediators, exacerbating the inflammatory state, and contributing to tissue damage in AITD (64, 65). The expression of IL-2ra is paramount for the proliferation and growth of T cells (66). Recent research has identified the occurrence of polymorphisms in this gene among patients with AITD (14), whereas Nakanishi et al. (67) observed elevated serum IL-2ra levels in patients with AITD. IL-4 is crucial for the development and function of helper Th2 cells (68). According to a previous study, the proportion of IL-4+ cells was increased within the thyroid tissues of HT patients (66), consistent with the results of our study. IL-17 serves as a primary effector of Th17 cells, playing a vital role in promoting their activation (69). Xue et al. (70) pointed out that the expression level of CD4+, IL-17+, and Th17 cells in the blood of untreated patients with AITD was higher than that of healthy individuals. Additionally, El-Zawawy et al. (71) reported a significant elevation in the expression level of serum IL-17A in patients with AITD compared to the healthy control group. IL-16 is a pro-inflammatory cytokine that exerts chemotactic effects on CD4 T lymphocytes, monocytes, and eosinophils, playing a role in both the inflammatory response and tumor development. Ongoing research has provided evidence of the expression of the IL-16 protein in the thyroid glands of patients diagnosed with GD and HT (72). Furthermore, existing evidence suggests that the expression levels of IL-16 in the serum could be a candidate marker for assessing disease activity and the severity of AITD (73).

To sum up, cytokines are involved in the pathogenesis of AITD (74). Their secretion profile can be either pro-inflammatory or anti-inflammatory and either pro-apoptotic or anti-apoptotic (75). These cytokines can participate in governing the immune system and the differentiation, growth, and secretory function of thyroid cells through their endocrine, autocrine, and paracrine modes. By modulating TSH levels, thyroid cells induce the abnormal expression of major histocompatibility complex–II antigens, which are transformed into antigen-presenting cells and resulting in autoimmune pathological damage and the development of AITD (76). Th1 lymphocytes generate pro-inflammatory proteins, including IFN-γ and IL-2, driving macrophage activation and inducing cytotoxic effects. On the other hand, Th2 lymphocytes generate anti-inflammatory proteins that suppress the production of Th1 cytokines. Additionally, they primarily stimulate B cells to generate antibodies and trigger the activation of anti-apoptotic molecules (77). Th17 lymphocytes release pro-inflammatory cytokines such as IL17 and exert a significant impact on persistent inflammation (78, 79).

While this study established a causal relationship and identified candidate targets for subsequent functional studies, it also has limitations that should not be overlooked. (1) This study used European population GWAS data for MR analysis, necessitating further studies in other populations. (2) Although MR is a highly efficient method for causality analysis, future animal tests are warranted to corroborate our findings. (3) The relationship between inflammatory cytokines and AITD is multifaceted, and elucidating the etiology and pathogenesis of circulating cytokines requires exploration from multiple aspects. (4) Ascribed to the complex etiology of AITD, circulating cytokines cannot fully explain the pathogenesis, necessitating more comprehensive data for further investigation.

In summary, the present study utilized the MR approach to investigate the causal association between inflammatory cytokines and AITD. Our results collectively unveiled that high levels of TNF-β and low levels of SCGF-β were associated with a high risk of developing GD. At the same time, high levels of IL-12p70, IL-13, and IFN-γ and low levels of MCP-1 and TNF-α suggest a higher risk of developing HD. Moreover, GD can increase the blood levels of MIP-1β, MCP-1, MIG, IP-10, SDF-1α, PDGFbb, βNGF, IL-2ra, IL-4, and IL-17, whereas HD can lead to elevated levels of MIG, IL-2ra, IP-10, and IL-16 levels. These cytokines hold significant implications for noninvasive early diagnosis of AITD. Finally, these cytokines may represent novel targets for the prevention, treatment, and long-term management of AITD.

## Data availability statement

The original contributions presented in the study are included in the article/**Supplementary Material**. Further inquiries can be directed to the corresponding authors.

## Ethics statement

Since the data used are publicly available in the database, no additional ethical approval was needed in this case.

## Author contributions

ZY: Investigation, Methodology, Project administration, Resources, Funding acquisition, Writing – review & editing. FG: Software, Supervision, Validation, Visualization, Writing – original draft. YT: Conceptualization, Methodology, Resources, Validation, Writing – original draft. YZ: Project administration, Resources, Software, Supervision, Writing – review & editing. YG: Conceptualization, Data curation, Project administration, Writing – original draft. GY: Data curation, Investigation, Project administration, Visualization, Writing – review & editing. SW: Conceptualization, Data curation, Formal analysis, Funding acquisition, Writing – original draft, Writing – review & editing.

## Supplementary material

Supplementary Table 1 The F number and R2 detect the intensity of the IVs between 41 Inflammatory cytokines and GD in MR analyses. GD, Graves’ Disease; IVs, instrumental variables.

Supplementary Table 2 The F number and R2 detect the intensity of the IVs between GD and 41 Inflammatory cytokines in MR analyses. GD, Graves’ Disease; IVs, instrumental variables.

Supplementary Table 3 The F number and R2 detect the intensity of the IVs between 41 Inflammatory cytokines and HT in MR analyses. HT, Hashimoto thyroiditis; IVs, instrumental variables.

Supplementary Table 4 The F number and R2 detect the intensity of the IVs between HT and 41 Inflammatory cytokines in MR analyses. HT, Hashimoto thyroiditis; IVs, instrumental variables.

## References

[1] Mammen JSR Cappola AR. Autoimmune thyroid disease in women. Jama. (2021) 325:2392–3. doi: 10.1001/jama.2020.22196 PMC10071442 33938930

[2] Conrad N Misra S Verbakel JY Verbeke G Molenberghs G Taylor PN. Incidence, prevalence, and co-occurrence of autoimmune disorders over time and by age, sex, and socioeconomic status: a population-based cohort study of 22 million individuals in the UK. Lancet. (2023) 401:1878–90. doi: 10.1016/s0140-6736(23)00457-9 37156255

[3] Antonelli A Ferrari SM Corrado A Di Domenicantonio A Fallahi P. Autoimmune thyroid disorders. Autoimmun Rev. (2015) 14:174–80. doi: 10.1016/j.autrev.2014.10.016 25461470

[4] Milo T Korem Kohanim Y Toledano Y Alon U. Autoimmune thyroid diseases as a cost of physiological autoimmune surveillance. Trends Immunol. (2023) 44:365–71. doi: 10.1016/j.it.2023.03.007 37061365

[5] Radziszewski M Kuś A Bednarczuk T. Genotype-phenotype correlations in Graves' disease. Best Pract Res Clin Endocrinol Metab. (2023) 37:101745. doi: 10.1016/j.beem.2023.101745 36828713

[6] Vargas-Uricoechea H. Molecular mechanisms in autoimmune thyroid disease. Cells. (2023) 12. doi: 10.3390/cells12060918 PMC10047067 36980259

[7] Liu C Chu D Kalantar-Zadeh K George J Young HA Liu G. Cytokines: from clinical significance to quantification. Adv Sci (Weinh). (2021) 8:e2004433. doi: 10.1002/advs.202004433 34114369 PMC8336501

[8] Jarczak D Nierhaus A. Cytokine storm-definition, causes, and implications. Int J Mol Sci. (2022) 23. doi: 10.3390/ijms231911740 PMC9570384 36233040

[9] Ferrari SM Paparo SR Ragusa F Elia G Mazzi V Patrizio A. Chemokines in thyroid autoimmunity. Best Pract Res Clin Endocrinol Metab. (2023) 37:101773. doi: 10.1016/j.beem.2023.101773 36907786

[10] Xiaoheng C Yizhou M Bei H Huilong L Xin W Rui H. General and specific genetic polymorphism of cytokines-related gene in AITD. Mediators Inflamm. (2017) 2017:3916395. doi: 10.1155/2017/3916395 28133421 PMC5241475

[11] Vasu C Holterman MJ Prabhakar BS. Modulation of dendritic cell function and cytokine production to prevent thyroid autoimmunity. Autoimmunity. (2003) 36:389–96. doi: 10.1080/08916930310001603073 14669946

[12] Li Q Wang B Mu K Zhang JA. The pathogenesis of thyroid autoimmune diseases: New T lymphocytes - Cytokines circuits beyond the Th1-Th2 paradigm. J Cell Physiol. (2019) 234:2204–16. doi: 10.1002/jcp.27180 30246383

[13] Wang Y Fang S Zhou H. Pathogenic role of Th17 cells in autoimmune thyroid disease and their underlying mechanisms. Best Pract Res Clin Endocrinol Metab. (2023) 37:101743. doi: 10.1016/j.beem.2023.101743 36841747

[14] Hwangbo Y Park YJ. Genome-wide association studies of autoimmune thyroid diseases, thyroid function, and thyroid cancer. Endocrinol Metab (Seoul). (2018) 33:175–84. doi: 10.3803/EnM.2018.33.2.175 PMC6021314 29947174

[15] Burgess S Daniel RM Butterworth AS Thompson SG. Network Mendelian randomization: using genetic variants as instrumental variables to investigate mediation in causal pathways. Int J Epidemiol. (2015) 44:484–95. doi: 10.1093/ije/dyu176 PMC4469795 25150977

[16] Lawlor DA Harbord RM Sterne JA Timpson N Davey Smith G. Mendelian randomization: using genes as instruments for making causal inferences in epidemiology. Stat Med. (2008) 27:1133–63. doi: 10.1002/sim.3034 17886233

[17] Ahola-Olli AV Würtz P Havulinna AS Aalto K Pitkänen N Lehtimäki T. Genome-wide association study identifies 27 loci influencing concentrations of circulating cytokines and growth factors. Am J Hum Genet. (2017) 100:40–50. doi: 10.1016/j.ajhg.2016.11.007 27989323 PMC5223028

[18] Sakaue S Kanai M Tanigawa Y Karjalainen J Kurki M Koshiba S. A cross-population atlas of genetic associations for 220 human phenotypes. Nat Genet. (2021) 53:1415–24. doi: 10.1038/s41588-021-00931-x PMC12208603 34594039

[19] Boehm FJ Zhou X. Statistical methods for Mendelian randomization in genome-wide association studies: A review. Comput Struct Biotechnol J. (2022) 20:2338–51. doi: 10.1016/j.csbj.2022.05.015 PMC9123217 35615025

[20] Burgess S Thompson SG. Interpreting findings from Mendelian randomization using the MR-Egger method. Eur J Epidemiol. (2017) 32:377–89. doi: 10.1007/s10654-017-0255-x PMC5506233 28527048

[21] Bowden J Davey Smith G Haycock PC Burgess S. Consistent estimation in mendelian randomization with some invalid instruments using a weighted median estimator. Genet Epidemiol. (2016) 40:304–14. doi: 10.1002/gepi.21965 PMC4849733 27061298

[22] Palmer TM Lawlor DA Harbord RM Sheehan NA Tobias JH Timpson NJ. Using multiple genetic variants as instrumental variables for modifiable risk factors. Stat Methods Med Res. (2012) 21:223–42. doi: 10.1177/0962280210394459 PMC3917707 21216802

[23] Meng C Deng P Miao R Tang H Li Y Wang J. Gut microbiome and risk of ischaemic stroke: a comprehensive Mendelian randomization study. Eur J Prev Cardiol. (2023) 30:613–20. doi: 10.1093/eurjpc/zwad052 36799937

[24] Greco MF Minelli C Sheehan NA Thompson JR. Detecting pleiotropy in Mendelian randomisation studies with summary data and a continuous outcome. Stat Med. (2015) 34:2926–40. doi: 10.1002/sim.6522 25950993

[25] Bowden J Davey Smith G Burgess S. Mendelian randomization with invalid instruments: effect estimation and bias detection through Egger regression. Int J Epidemiol. (2015) 44:512–25. doi: 10.1093/ije/dyv080 PMC4469799 26050253

[26] Hinck AP Mueller TD Springer TA. Structural biology and evolution of the TGF-β Family. Cold Spring Harb Perspect Biol. (2016) 8. doi: 10.1101/cshperspect.a022103 PMC5131774 27638177

[27] Bidey SP Hill DJ Eggo MC. Growth factors and goitrogenesis. J Endocrinol. (1999) 160:321–32. doi: 10.1677/joe.0.1600321 10076179

[28] Stassi G De Maria R. Autoimmune thyroid disease: new models of cell death in autoimmunity. Nat Rev Immunol. (2002) 2:195–204. doi: 10.1038/nri750 11913070

[29] Vander Ark A Cao J Li X. TGF-β receptors: In and beyond TGF-β signaling. Cell Signal. (2018) 52:112–20. doi: 10.1016/j.cellsig.2018.09.002 30184463

[30] Derynck R Zhang YE. Smad-dependent and Smad-independent pathways in TGF-beta family signalling. Nature. (2003) 425:577–84. doi: 10.1038/nature02006 14534577

[31] Widder J Dorfinger K Wilfing A Trieb K Pirich K Loebenstein R. The immunoregulatory influence of transforming growth factor beta in thyroid autoimmunity: TGF beta inhibits autoreactivity in Graves' disease. J Autoimmun. (1991) 4:689–701. doi: 10.1016/0896-8411(91)90186-g 1777015

[32] Schiro A Wilkinson FL Weston R Smyth JV Serracino-Inglott F Alexander MY. Elevated levels of endothelial-derived microparticles, and serum CXCL9 and SCGF-β are associated with unstable asymptomatic carotid plaques. Sci Rep. (2015) 5:16658. doi: 10.1038/srep16658 26564003 PMC4643236

[33] Chen Z Hu Z Hu Y Sheng Y Li Y Song J. Novel potential biomarker of adult cardiac surgery-associated acute kidney injury. Front Physiol. (2020) 11:587204. doi: 10.3389/fphys.2020.587204 33240107 PMC7683426

[34] Vignali DA Kuchroo VK. IL-12 family cytokines: immunological playmakers. Nat Immunol. (2012) 13:722–8. doi: 10.1038/ni.2366 PMC4158817 22814351

[35] Inoue N Watanabe M Nakaguchi A Ueda D Kawaguti H Hidaka Y. Functional polymorphisms affecting Th1 differentiation are associated with the severity of autoimmune thyroid diseases. Endocr J. (2017) 64:695–703. doi: 10.1507/endocrj.EJ16-0551 28515387

[36] Zhang W Flynn JC Kong YC. IL-12 prevents tolerance induction with mouse thyroglobulin by priming pathogenic T cells in experimental autoimmune thyroiditis: role of IFN-gamma and the costimulatory molecules CD40l and CD28. Cell Immunol. (2001) 208:52–61. doi: 10.1006/cimm.2001.1767 11277619

[37] Matera MG Ora J Calzetta L Rogliani P Cazzola M. Investigational anti IL-13 asthma treatments: a 2023 update. Expert Opin Investig Drugs. (2023) 32:373–86. doi: 10.1080/13543784.2023.2215425 37194672

[38] Shankar A McAlees JW Lewkowich IP. Modulation of IL-4/IL-13 cytokine signaling in the context of allergic disease. J Allergy Clin Immunol. (2022) 150:266–76. doi: 10.1016/j.jaci.2022.06.012 PMC9371363 35934680

[39] Thuner J Coutant F. IFN-γ: An overlooked cytokine in dermatomyositis with anti-MDA5 antibodies. Autoimmun Rev. (2023) 22:103420. doi: 10.1016/j.autrev.2023.103420 37625674

[40] Salzano M Russo E Postiglione L Guerra A Marotta V Esposito S. Interferon-γ inhibits integrin-mediated adhesion to fibronectin and survival signaling in thyroid cells. J Endocrinol. (2012) 215:439–44. doi: 10.1530/joe-12-0335 23027608

[41] Bossowski A Harasymczuk J Moniuszko A Bossowska A Hilczer M Ratomski K. Cytometric evaluation of intracellular IFN-γ and IL-4 levels in thyroid follicular cells from patients with autoimmune thyroid diseases. Thyroid Res. (2011) 4:13. doi: 10.1186/1756-6614-4-13 21943174 PMC3189885

[42] Emi Aikawa N de Carvalho JF Artur Almeida Silva C Bonfá E. Immunogenicity of Anti-TNF-alpha agents in autoimmune diseases. Clin Rev Allergy Immunol. (2010) 38:82–9. doi: 10.1007/s12016-009-8140-3 19565360

[43] Jin J Chang Y Wei W. Clinical application and evaluation of anti-TNF-alpha agents for the treatment of rheumatoid arthritis. Acta Pharmacol Sin. (2010) 31:1133–40. doi: 10.1038/aps.2010.134 PMC4002310 20711219

[44] van Hogezand RA Verspaget HW. The future role of anti-tumour necrosis factor-alpha products in the treatment of Crohn's disease. Drugs. (1998) 56:299–305. doi: 10.2165/00003495-199856030-00001 9777308

[45] Singh S Anshita D Ravichandiran V. MCP-1: Function, regulation, and involvement in disease. Int Immunopharmacol. (2021) 101:107598. doi: 10.1016/j.intimp.2021.107598 34233864 PMC8135227

[46] Kemp EH Metcalfe RA Smith KA Woodroofe MN Watson PF Weetman AP. Detection and localization of chemokine gene expression in autoimmune thyroid disease. Clin Endocrinol (Oxf). (2003) 59:207–13. doi: 10.1046/j.1365-2265.2003.01824.x 12864798

[47] Ozisik H Cekin A Suner A Durmaz B Ozel B Gunel NS. Evaluation of IL-10, MCP-1, IFN gamma, and protectin D1 levels in patients with Hashimoto’s thyroiditis. Ir J Med Sci. (2023) 192:177–84. doi: 10.1007/s11845-022-03231-3 36434424

[48] Maurer M von Stebut E. Macrophage inflammatory protein-1. Int J Biochem Cell Biol. (2004) 36:1882–6. doi: 10.1016/j.biocel.2003.10.019 15203102

[49] Irving SG Zipfel PF Balke J McBride OW Morton CC Burd PR. Two inflammatory mediator cytokine genes are closely linked and variably amplified on chromosome 17q. Nucleic Acids Res. (1990) 18:3261–70. doi: 10.1093/nar/18.11.3261 PMC330932 1972563

[50] Ferrari SM Fallahi P Elia G Ragusa F Camastra S Paparo SR. Novel therapies for thyroid autoimmune diseases: An update. Best Pract Res Clin Endocrinol Metab. (2020) 34:101366. doi: 10.1016/j.beem.2019.101366 31813786

[51] Rotondi M Chiovato L. The chemokine system as a therapeutic target in autoimmune thyroid diseases: a focus on the interferon-γ inducible chemokines and their receptor. Curr Pharm Des. (2011) 17:3202–16. doi: 10.2174/138161211798157559 21864266

[52] Loetscher M Gerber B Loetscher P Jones SA Piali L Clark-Lewis I. Chemokine receptor specific for IP10 and mig: structure, function, and expression in activated T-lymphocytes. J Exp Med. (1996) 184:963–9. doi: 10.1084/jem.184.3.963 PMC2192763 9064356

[53] García-López MA Sancho D Sánchez-Madrid F Marazuela M. Thyrocytes from autoimmune thyroid disorders produce the chemokines IP-10 and Mig and attract CXCR3+ lymphocytes. J Clin Endocrinol Metab. (2001) 86:5008–16. doi: 10.1210/jcem.86.10.7953 11600578

[54] Romagnani P Rotondi M Lazzeri E Lasagni L Francalanci M Buonamano A. Expression of IP-10/CXCL10 and MIG/CXCL9 in the thyroid and increased levels of IP-10/CXCL10 in the serum of patients with recent-onset Graves’ disease. Am J Pathol. (2002) 161:195–206. doi: 10.1016/s0002-9440(10)64171-5 12107104 PMC1850693

[55] Ghadge SK Mühlstedt S Ozcelik C Bader M. SDF-1α as a therapeutic stem cell homing factor in myocardial infarction. Pharmacol Ther. (2011) 129:97–108. doi: 10.1016/j.pharmthera.2010.09.011 20965212

[56] Meng Z Feng G Hu X Yang L Yang X Jin Q. SDF factor-1α Promotes the migration, proliferation, and osteogenic differentiation of mouse bone marrow mesenchymal stem cells through the wnt/β-catenin pathway. Stem Cells Dev. (2021) 30:106–17. doi: 10.1089/scd.2020.0165 33234049

[57] Werner TA Forster CM Dizdar L Verde PE Raba K Schott M. CXCR4/CXCR7/CXCL12-axis in follicular thyroid carcinoma. J Cancer. (2018) 9:929–40. doi: 10.7150/jca.23042 PMC5868160 29581772

[58] Castellone MD Guarino V De Falco V Carlomagno F Basolo F Faviana P. Functional expression of the CXCR4 chemokine receptor is induced by RET/PTC oncogenes and is a common event in human papillary thyroid carcinomas. Oncogene. (2004) 23:5958–67. doi: 10.1038/sj.onc.1207790 15184868

[59] Hoch RV Soriano P. Roles of PDGF in animal development. Development. (2003) 130:4769–84. doi: 10.1242/dev.00721 12952899

[60] Andrae J Gallini R Betsholtz C. Role of platelet-derived growth factors in physiology and medicine. Genes Dev. (2008) 22:1276–312. doi: 10.1101/gad.1653708 PMC2732412 18483217

[61] Virakul S van Steensel L Dalm VA Paridaens D van Hagen PM Dik WA. Platelet-derived growth factor: a key factor in the pathogenesis of graves' ophthalmopathy and potential target for treatment. Eur Thyroid J. (2014) 3:217–26. doi: 10.1159/000367968 PMC4311307 25759797

[62] Berry A Bindocci E Alleva E. NGF, brain and behavioral plasticity. Neural Plast. (2012) 2012:784040. doi: 10.1155/2012/784040 22474604 PMC3306960

[63] Terracina S Ferraguti G Tarani L Fanfarillo F Tirassa P Ralli M. Nerve growth factor and autoimmune diseases. Curr Issues Mol Biol. (2023) 45:8950–73. doi: 10.3390/cimb45110562 PMC10670231 37998739

[64] Dicou E Nerrière V. Evidence that natural autoantibodies against the nerve growth factor (NGF) may be potential carriers of NGF. J Neuroimmunol. (1997) 75:200–3. doi: 10.1016/s0165-5728(97)00008-8 9143255

[65] Dicou E Masson C Jabbour W Nerriere V. Increased frequency of NGF in sera of rheumatoid arthritis and systemic lupus erythematosus patients. Neuroreport. (1993) 5:321–4. doi: 10.1097/00001756-199312000-00036 7507728

[66] Zwirska-Korczala K Berdowska A Jochem J Sitkiewicz A Birkner E Polaniak R. Influence of thyroxine on serum soluble interleukin-2 receptor alpha levels in thyroid disorders. J Clin Pharm Ther. (2004) 29:151–6. doi: 10.1111/j.1365-2710.2004.00547.x 15068404

[67] Chistiakov DA Chistiakova EI Voronova NV Turakulov RI Savost’anov KV. A variant of the Il2ra / Cd25 gene predisposing to graves’ disease is associated with increased levels of soluble interleukin-2 receptor. Scand J Immunol. (2011) 74:496–501. doi: 10.1111/j.1365-3083.2011.02608.x 21815908

[68] Keegan AD Zamorano J. Regulation of gene expression, growth, and cell survival by IL-4: contribution of multiple signaling pathways. Cell Res. (1998) 8:1–13. doi: 10.1038/cr.1998.1 9570012

[69] Huangfu L Li R Huang Y Wang S. The IL-17 family in diseases: from bench to bedside. Signal Transduct Target Ther. (2023) 8:402. doi: 10.1038/s41392-023-01620-3 37816755 PMC10564932

[70] Xue H Yu X Ma L Song S Li Y Zhang L. The possible role of CD4^+^CD25(high)Foxp3^+^/CD4^+^IL-17A^+^ cell imbalance in the autoimmunity of patients with Hashimoto thyroiditis. Endocrine. (2015) 50:665–73. doi: 10.1007/s12020-015-0569-y 25771887

[71] El-Zawawy HT Farag HF Tolba MM Abdalsamea HA. Improving Hashimoto's thyroiditis by eradicating Blastocystis hominis: Relation to IL-17. Ther Adv Endocrinol Metab. (2020) 11:2042018820907013. doi: 10.1177/2042018820907013 32128107 PMC7036484

[72] Kemp EH Ajjan RA Metcalfe RA Watson PF Weetman AP. IL-14 and IL-16 are expressed in the thyroid of patients with either Graves’ disease or Hashimoto’s thyroiditis. Clin Endocrinol (Oxf). (2015) 83:726–32. doi: 10.1111/cen.12810 25940130

[73] Gu X Zheng L Chen X Ruan L Zhang H Ge S. Elevated serum IL-16 and RANTES levels in patients with autoimmune thyroid diseases and modulation by methimazole therapy. Horm Metab Res. (2012) 44:482–7. doi: 10.1055/s-0032-1308973 22473756

[74] Ferrari SM Fallahi P Elia G Ragusa F Ruffilli I Paparo SR. Thyroid autoimmune disorders and cancer. Semin Cancer Biol. (2020) 64:135–46. doi: 10.1016/j.semcancer.2019.05.019 31158464

[75] Ramani T Auletta CS Weinstock D Mounho-Zamora B Ryan PC Salcedo TW. Cytokines: the good, the bad, and the deadly. Int J Toxicol. (2015) 34:355–65. doi: 10.1177/1091581815584918 26015504

[76] Kong YC Lomo LC Motte RW Giraldo AA Baisch J Strauss G. HLA-DRB1 polymorphism determines susceptibility to autoimmune thyroiditis in transgenic mice: definitive association with HLA-DRB1*0301 (DR3) gene. J Exp Med. (1996) 184:1167–72. doi: 10.1084/jem.184.3.1167 PMC2192804 9064334

[77] Berger A. Th1 and Th2 responses: what are they? Bmj. (2000) 321:424. doi: 10.1136/bmj.321.7258.424 10938051 PMC27457

[78] Prud'homme GJ Piccirillo CA. The inhibitory effects of transforming growth factor-beta-1 (TGF-beta1) in autoimmune diseases. J Autoimmun. (2000) 14:23–42. doi: 10.1006/jaut.1999.0339 10648114

[79] Nanba T Watanabe M Inoue N Iwatani Y. Increases of the Th1/Th2 cell ratio in severe Hashimoto’s disease and in the proportion of Th17 cells in intractable Graves' disease. Thyroid. (2009) 19:495–501. doi: 10.1089/thy.2008.0423 19415997

