# Supplementary material for: Causal relationship between inflammatory cytokines and autoimmune thyroid disease: a bidirectional two-sample Mendelian randomization analysis
Source: Front Immunol. 2024 Mar 20;15:1334772. doi: 10.3389/fimmu.2024.1334772 (PMC10989681; doi:10.3389/fimmu.2024.1334772)
Supplement: Supplementary file 5 [file Image_1.pdf]

Supplementary Figures

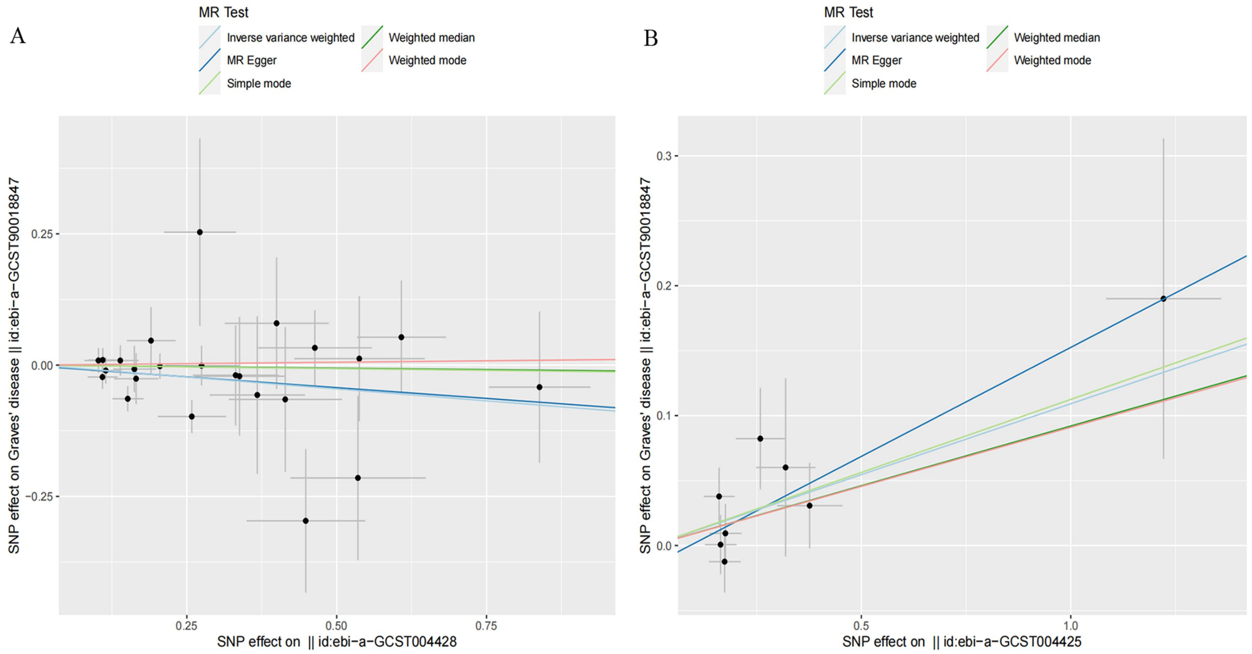

Supplementary Figure 1. Scatter plots of Mendelian randomization analyses for SCGFβ(A), and TNFβ (B) in GD.

Abbreviations: GD, Graves' Disease; SNP, Single nucleotide polymorphism; SCGF β , Stem cell growth factor beta; TNF β , Tumor necrosis factor-beta.

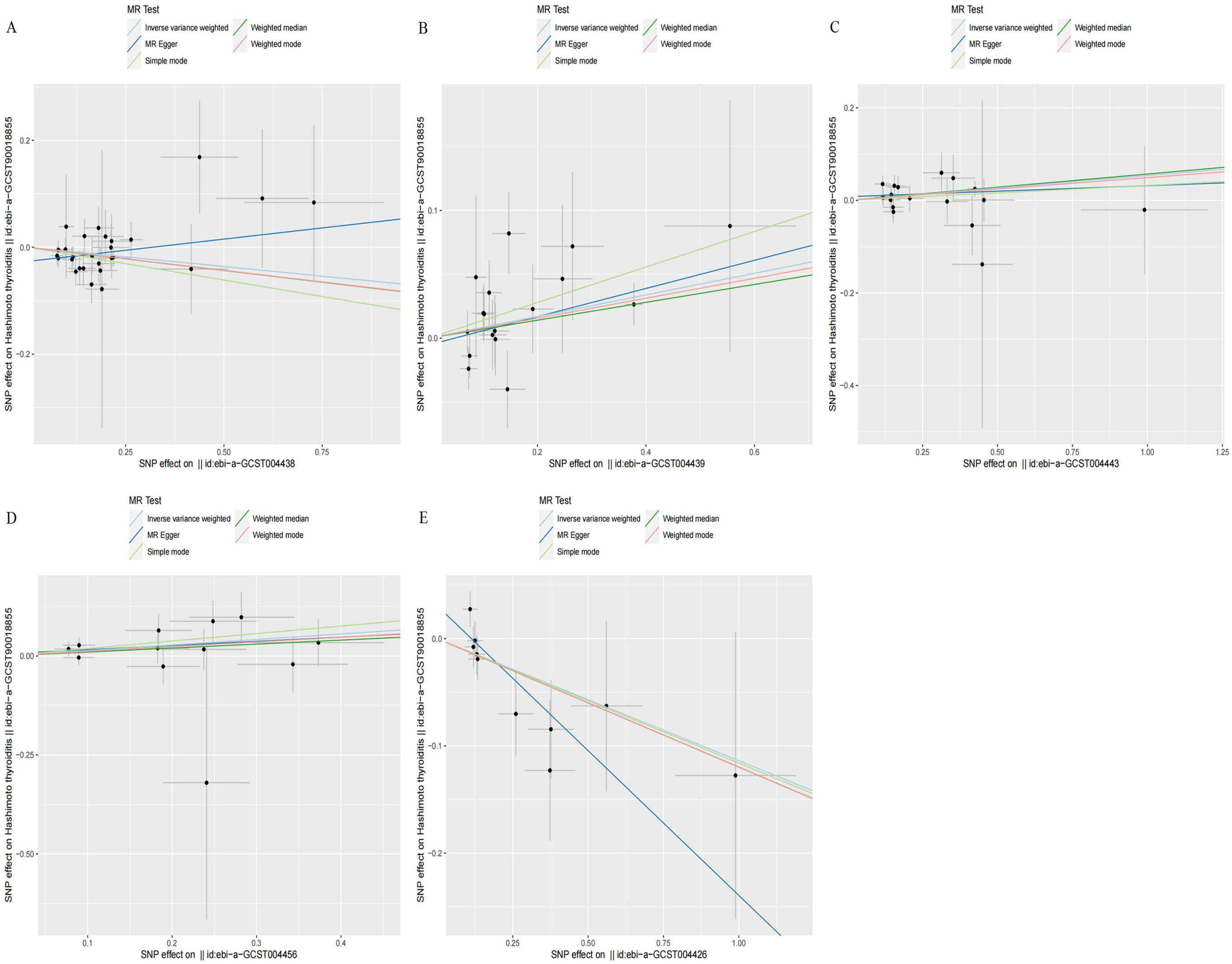

Supplementary Figure 2. Scatter plots of Mendelian randomization analyses between inflammatory cytokines (A-E: MCP1, IL-12p70, IL-13, IFN-γ and TNFα) and HT.

Abbreviations: MCP1, Monocyte chemotactic protein-1; IL-12p70, Interleukin-12p70; IL-13, Interleukin-13; IFN-γ ,Interferon-gamma; TNFα , Tumor necrosis factor-alpha; HT, Hashimoto thyroiditis.

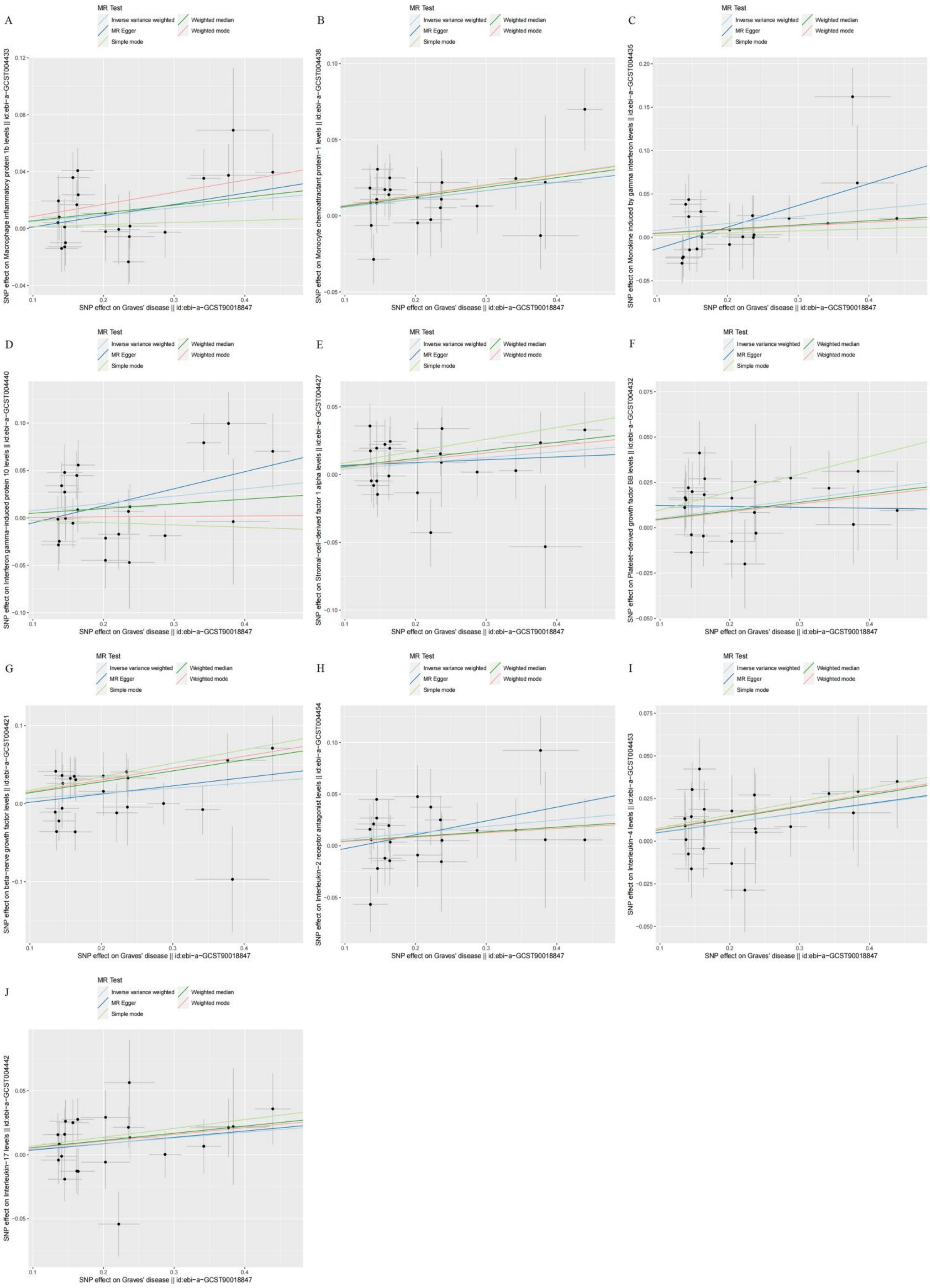
A
MR Test
Inverse variance weighted
MR Egger
Simple mode
Weighted median
Weighted mode
SNP effect on Macrophage inflammatory protein 1b levels || id:ebi−a−GCST004433
SNP effect on Graves' disease || id:ebi−a−GCST90018847
B
SNP effect on Monocyte chemoattractant protein−1 levels || id:ebi−a−GCST004438
C
SNP effect on Monokine induced by gamma interferon levels || id:ebi−a−GCST004435
D
SNP effect on Interferon gamma−induced protein 10 levels || id:ebi−a−GCST004440
E
SNP effect on Stromal−cell−derived factor 1 alpha levels || id:ebi−a−GCST004427
F
SNP effect on Platelet−derived growth factor BB levels || id:ebi−a−GCST004432
G
SNP effect on beta−nerve growth factor levels || id:ebi−a−GCST004421
H
SNP effect on Interleukin−2 receptor antagonist levels || id:ebi−a−GCST004454
I
SNP effect on Interleukin−4 levels || id:ebi−a−GCST004453
J
SNP effect on Interleukin−17 levels || id:ebi−a−GCST004442

Supplementary Figure 3. Scatter plots of Mendelian randomization analyses between GD and inflammatory cytokines (A-J: MIP1β, MIP1, MIG, IP10, SDF1α, PDGFbb, βNGF, IL2ra, IL-4 and IL-17).

Abbreviations: GD, Graves' Disease; SNP, Single nucleotide polymorphism;MIP1β,Macrophage infammatory protein-1β; MIP1,Monocyte chemotactic protein-1; MIG,Monokine induced by interferon-gamma; IP10, Interferon gamma-induced protein 10; SDF1α, Stromal cell-derived factor-1 alpha; PDGFbb,Platelet derived growth factor BB; βNGF,Beta nerve growth factor; IL2ra,Interleukin-2 receptor, alpha subunit; IL-4, Interleukin-4 ;IL-17,Interleukin-17.

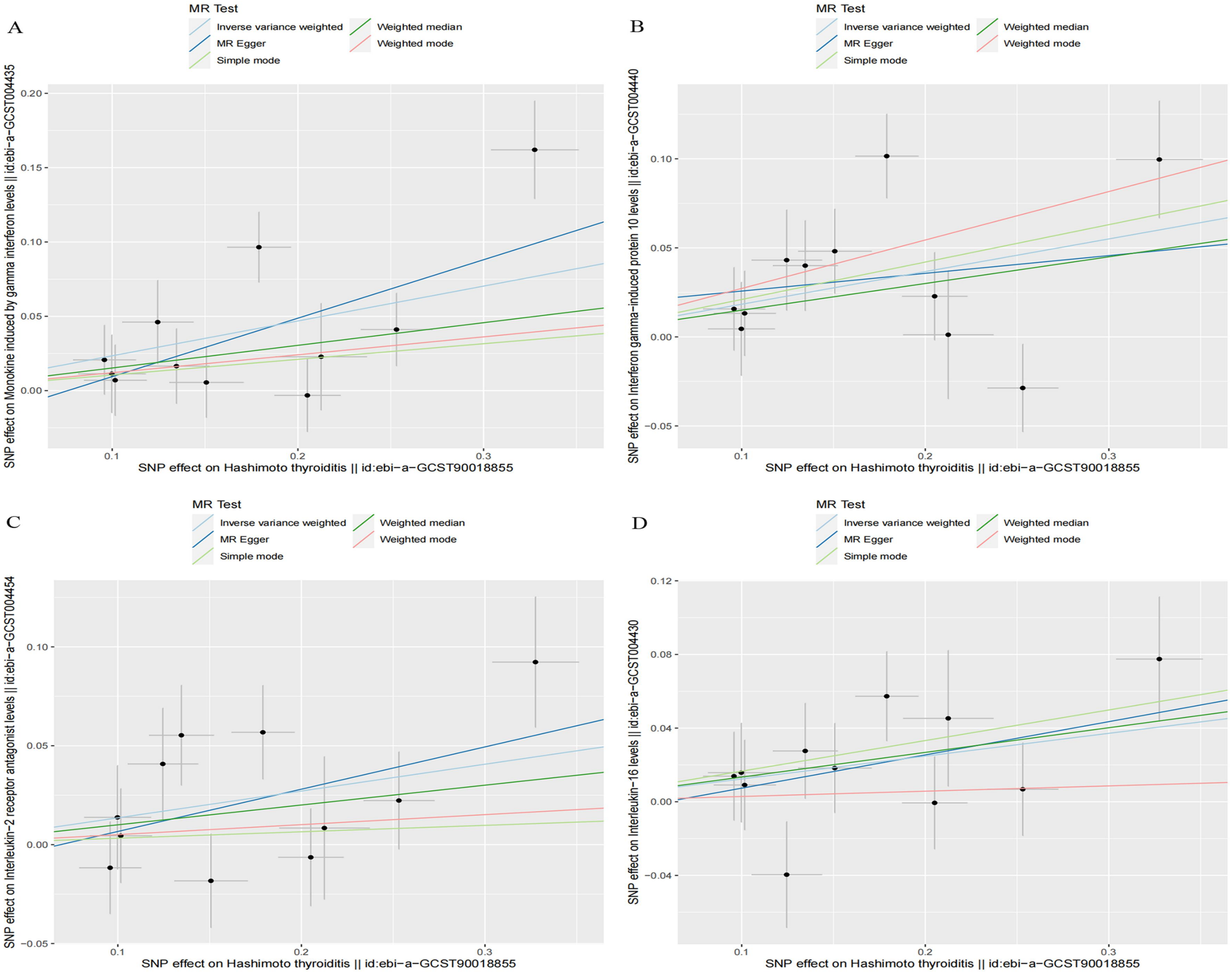

Supplementary Figure 4. Scatter plots of Mendelian randomization analyses between HT and inflammatory cytokines (A-D: MIG, IP10, IL2ra and IL-16).

Abbreviations: HT, Hashimoto thyroiditis.; MIG,Monokine induced by interferon-gamma; IP10, Interferon gamma-induced protein 10; IL2ra,Interleukin-2 receptor, alpha subunit;IL-16,Interleukin-16.

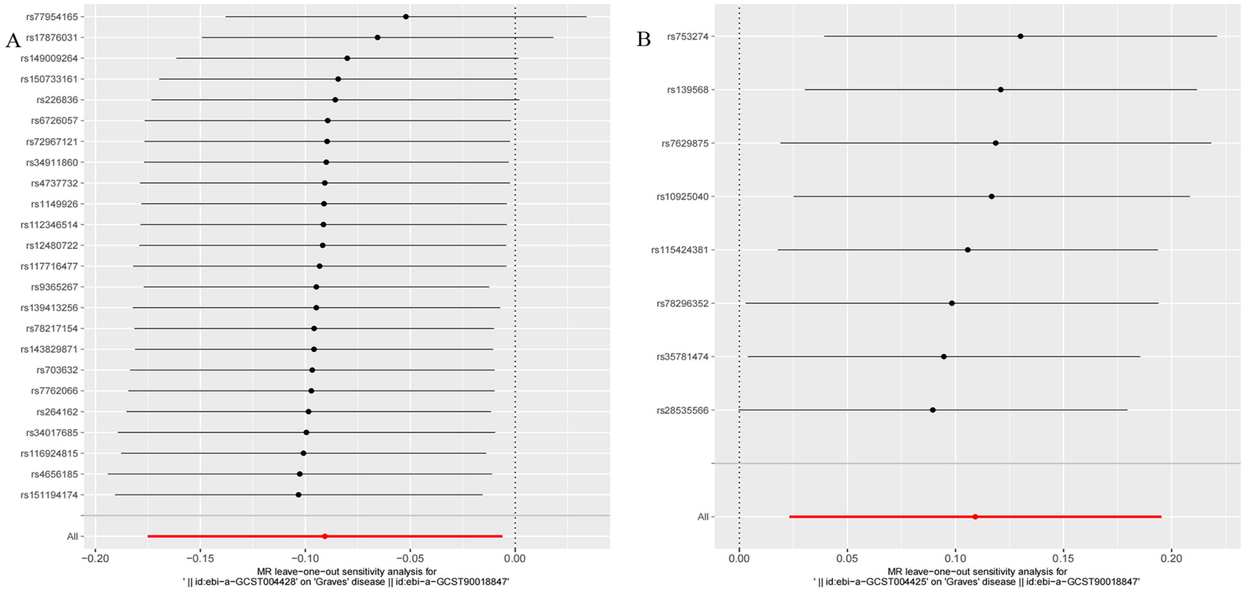

Supplementary Figure 5. Leave-one-out sensitivity analyses of Mendelian randomization analyses for SCGFβ(A), and TNFβ (B) in GD.

Abbreviations: GD, Graves' Disease; SCGF β , Stem cell growth factor beta; TNF β , Tumor necrosis factor-beta.

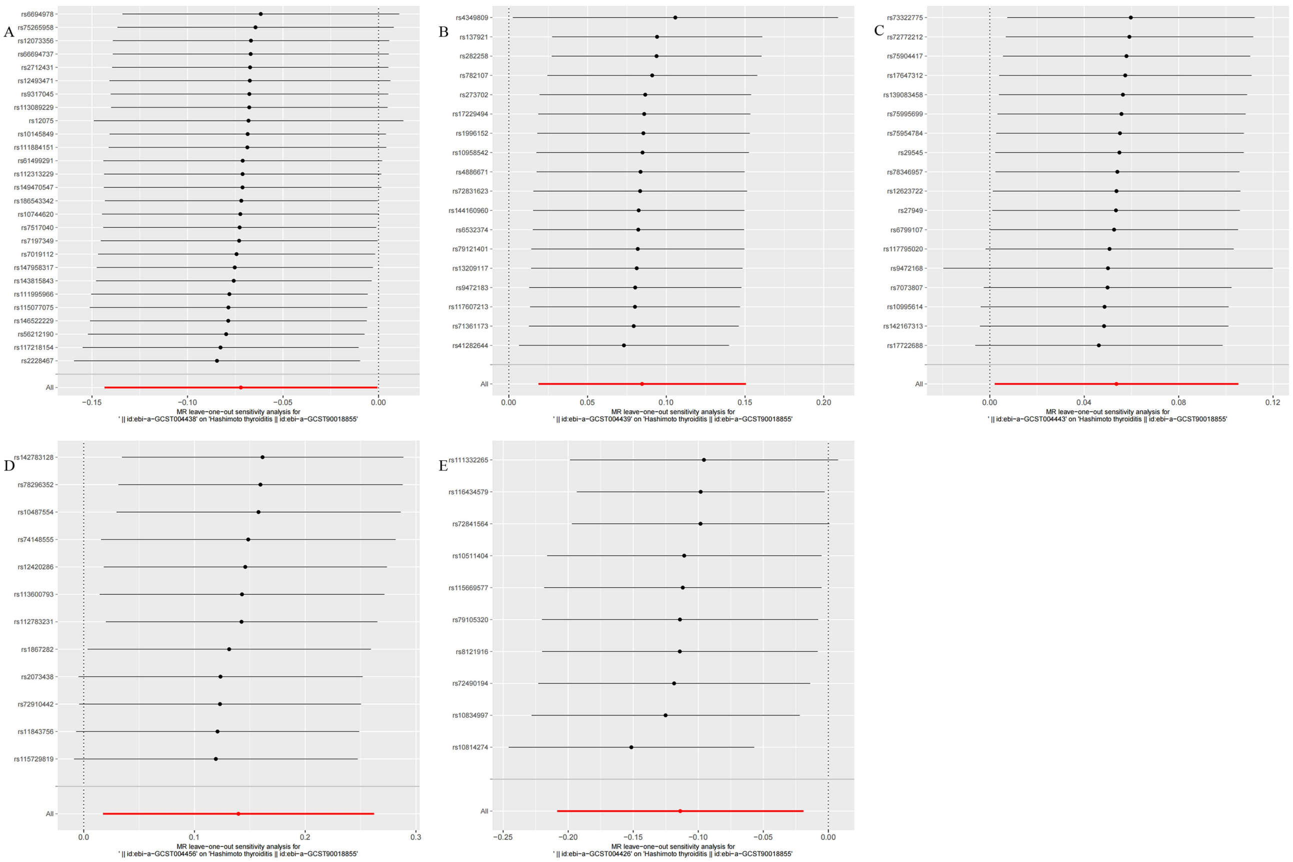

Supplementary Figure 6. Leave-one-out sensitivity analyses of Mendelian randomization analyses for inflammatory cytokines (A-E: MCP1, IL-12p70, IL-13, IFN-γ and TNFα) in HT.

Abbreviations: MCP1, Monocyte chemotactic protein-1; IL-12p70, Interleukin-12p70; IL-13, Interleukin-13; IFN-γ ,Interferon-gamma; TNFα, Tumor necrosis factor-alpha; HT, Hashimoto thyroiditis.

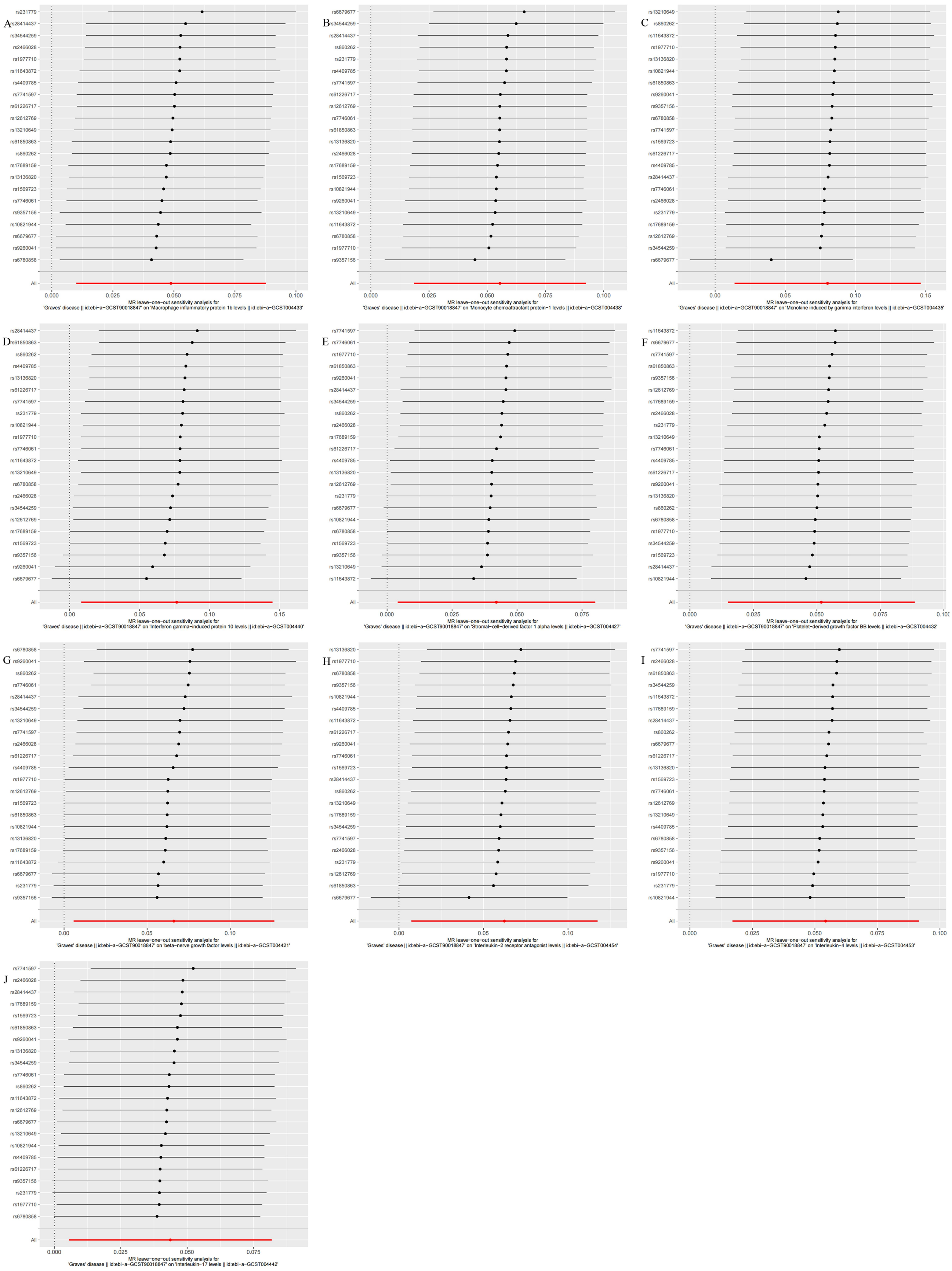
A
MR leave−one−out sensitivity analysis for
'Graves' disease || id:ebi−a−GCST90018847' on 'Macrophage inflammatory protein 1b levels || id:ebi−a−GCST004433'
B
MR leave−one−out sensitivity analysis for
'Graves' disease || id:ebi−a−GCST90018847' on 'Monocyte chemoattractant protein−1 levels || id:ebi−a−GCST004438'
C
MR leave−one−out sensitivity analysis for
'Graves' disease || id:ebi−a−GCST90018847' on 'Monokine induced by gamma interferon levels || id:ebi−a−GCST004435'
D
MR leave−one−out sensitivity analysis for
'Graves' disease || id:ebi−a−GCST90018847' on 'Interferon gamma−induced protein 10 levels || id:ebi−a−GCST004440'
E
MR leave−one−out sensitivity analysis for
'Graves' disease || id:ebi−a−GCST90018847' on 'Stromal−cell−derived factor 1 alpha levels || id:ebi−a−GCST004427'
F
MR leave−one−out sensitivity analysis for
'Graves' disease || id:ebi−a−GCST90018847' on 'Platelet−derived growth factor BB levels || id:ebi−a−GCST004432'
G
MR leave−one−out sensitivity analysis for
'Graves' disease || id:ebi−a−GCST90018847' on 'beta−nerve growth factor levels || id:ebi−a−GCST004421'
H
MR leave−one−out sensitivity analysis for
'Graves' disease || id:ebi−a−GCST90018847' on 'Interleukin−2 receptor antagonist levels || id:ebi−a−GCST004454'
I
MR leave−one−out sensitivity analysis for
'Graves' disease || id:ebi−a−GCST90018847' on 'Interleukin−4 levels || id:ebi−a−GCST004453'
J
MR leave−one−out sensitivity analysis for
'Graves' disease || id:ebi−a−GCST90018847' on 'Interleukin−17 levels || id:ebi−a−GCST004442'

Supplementary Figure 7. Leave-one-out sensitivity analyses of Mendelian randomization analyses between GD and inflammatory cytokines (A-J: MIP1 β , MIP1, MIG, IP10, SDF1 α , PDGFbb, β NGF, IL2ra, IL-4 and IL-17).

Abbreviations: GD, Graves' Disease; SNP, Single nucleotide polymorphism;MIP1 β ,Macrophage infammatory protein-1 β ; MIP1,Monocyte chemotactic protein-1; MIG,Monokine induced by interferon-gamma; IP10, Interferon gamma-induced protein 10; SDF1 α , Stromal cell-derived factor-1 alpha; PDGFbb,Platelet derived growth factor BB; β NGF,Beta nerve growth factor; IL2ra,Interleukin-2 receptor, alpha subunit; IL-4, Interleukin-4 ;IL-17,Interleukin-17.

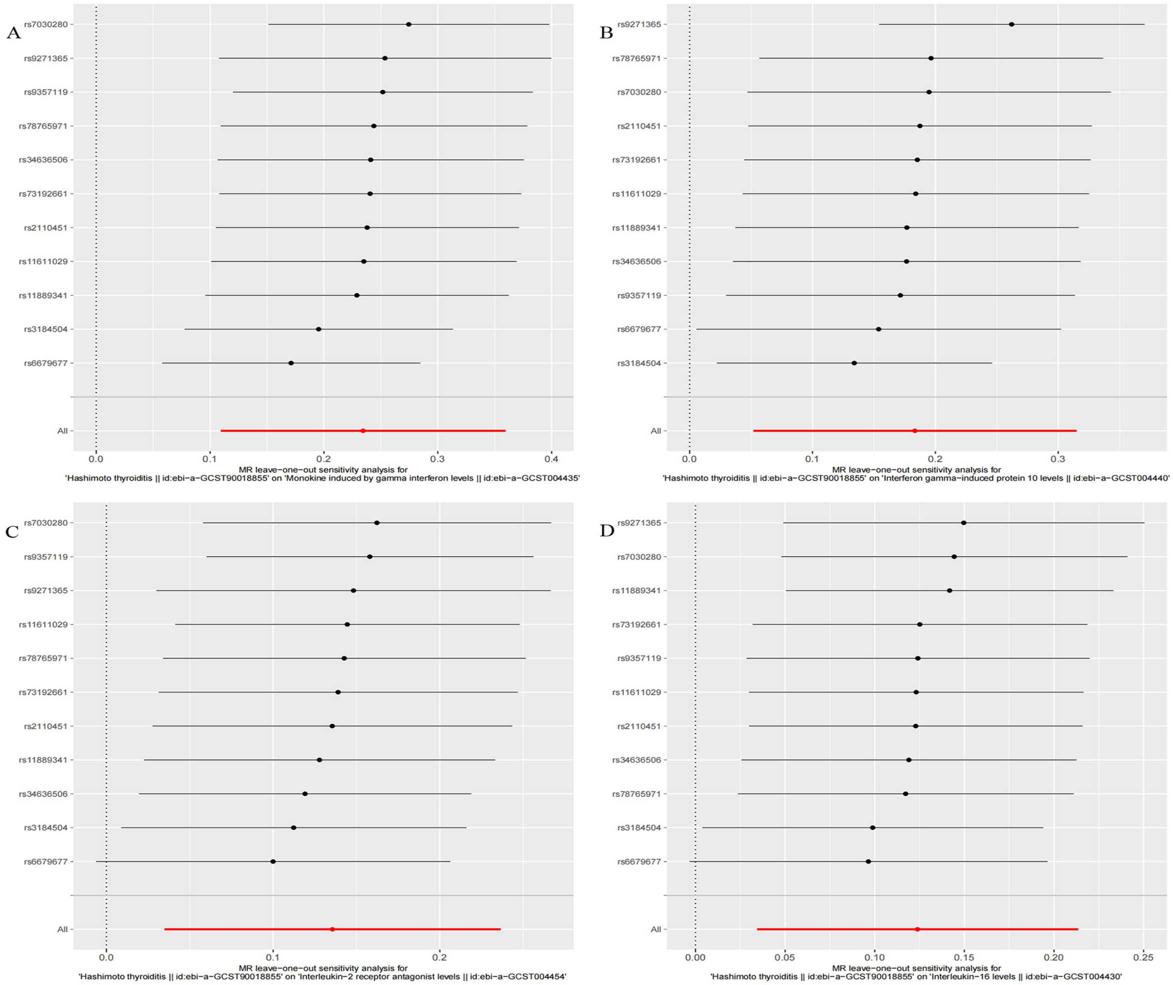

Supplementary Figure 8. Leave-one-out sensitivity analyses of Mendelian randomization analyses between HT and inflammatory cytokines (A-D: MIG, IP10, IL2ra and IL-16).

Abbreviations: HT, Hashimoto thyroiditis.; MIG,Monokine induced by interferon-gamma; IP10, Interferon gamma-induced protein 10; IL2ra,Interleukin-2 receptor, alpha subunit;IL-16,Interleukin-16.
